# Evidence for Autoregulation and Cell Signaling Pathway Regulation From Genome-Wide Binding of the *Drosophila* Retinoblastoma Protein

**DOI:** 10.1534/g3.112.004424

**Published:** 2012-11-01

**Authors:** Pankaj Acharya, Nicolas Negre, John Johnston, Yiliang Wei, Kevin P. White, R. William Henry, David N. Arnosti

**Affiliations:** * Department of Microbiology and Molecular Genetics, Michigan State University, East Lansing, Michigan 48824-1319; ‡ Department of Biochemistry and Molecular Biology, Michigan State University, East Lansing, Michigan 48824-1319; † Institute for Genomics and Systems Biology, University of Chicago, Chicago, Illinois 60637

**Keywords:** retinoblastoma, Rbf1, cell-cycle, Drosophila

## Abstract

The retinoblastoma (RB) tumor suppressor protein is a transcriptional cofactor with essential roles in cell cycle and development. Physical and functional targets of RB and its paralogs p107/p130 have been studied largely in cultured cells, but the full biological context of this family of proteins’ activities will likely be revealed only in whole organismal studies. To identify direct targets of the major Drosophila RB counterpart in a developmental context, we carried out ChIP-Seq analysis of Rbf1 in the embryo. The association of the protein with promoters is developmentally controlled; early promoter access is globally inhibited, whereas later in development Rbf1 is found to associate with promoter-proximal regions of approximately 2000 genes. In addition to conserved cell-cycle–related genes, a wholly unexpected finding was that Rbf1 targets many components of the insulin, Hippo, JAK/STAT, Notch, and other conserved signaling pathways. Rbf1 may thus directly affect output of these essential growth-control and differentiation pathways by regulation of expression of receptors, kinases and downstream effectors. Rbf1 was also found to target multiple levels of its own regulatory hierarchy. Bioinformatic analysis indicates that different classes of genes exhibit distinct constellations of motifs associated with the Rbf1-bound regions, suggesting that the context of Rbf1 recruitment may vary within the Rbf1 regulon. Many of these targeted genes are bound by Rbf1 homologs in human cells, indicating that a conserved role of RB proteins may be to adjust the set point of interlinked signaling networks essential for growth and development.

The retinoblastoma tumor suppressor protein (RB) is an evolutionarily conserved transcriptional corepressor that controls cell-cycle differentiation, development, autophagy, and apoptosis (Lipinski and Jacks 1999; Bosco *et al.* 2001; Nevins 2001; Classon and Harlow 2002; Jiang *et al.* 2010). Germline mutations of RB are closely linked to retinoblastoma in early childhood and osteosarcoma in adolescence, and somatic mutations in the RB gene are extremely frequent in human cancers (Lohmann 1999; Sherr and McCormick 2002; Tang *et al.* 2008). The vertebrate RB protein and the related family members, p107 and p130, are recruited to promoters by interactions with E2F/DP heterodimers (van den Heuvel and Dyson 2008). Interactions between E2F transcription factors and RB family proteins are regulated by cyclin/CDK-directed phosphorylation during the cell cycle, and RB-E2F interactions can also be affected by viral proteins (Nevins 1992a,b; Dick 2007; Flowers *et al.* 2010). Although less complex than its human counterpart, the *Drosophila* retinoblastoma network is functionally conserved and consists of two RB proteins, Rbf1 and Rbf2, two E2F proteins, E2F1 and E2F2, and one DP protein (Du and Pogoriler 2006). *Drosophila* Rbf proteins are regulated by phosphorylation, similar to the vertebrate RB proteins (Xin *et al.* 2002; Frolov *et al.* 2005). Rbf1 activity is also regulated during development by proteosome-dependent degradation, which is dependent on a C-terminal instability element that is simultaneously required for corepressor activity (Acharya *et al.* 2010). The instability mechanism is conserved in the p107 human homolog, indicating that this novel linkage between protein lability and repression function may be a general property of these proteins in multicellular organisms (Acharya *et al.* 2010).

RB proteins are involved in regulation of both canonical and noncanonical forms of the cell cycle. The canonical cycling involves the separation of DNA-synthesis (S-phase) and mitosis (M-phase) by two gap phases, G1 and G2. However, during development, noncanonical mitotic programs are common. In *Drosophila*, the first 13 embryonic cell cycles are synchronous, consisting of only S and M phases. G2 appears in cell cycle 14 and G1 in cell cycle 17 (Foe 1989; Edgar and O’Farrell 1990; Shibutani *et al.* 2007). Endoreplication (in which mitosis is not followed by cytokinesis) is another variant common in many larval and adult tissues (Spradling and Orr-Weaver 1987). Endoreduplication in follicle cells is regulated by Rbf1/E2F (Bosco *et al.* 2001). This diversity of cell-cycle regulation suggests that Rbf1/2 and its partners may be differentially used or regulated in different settings. Consistent with this idea, Rbf1 stability is decreased in proliferating larval imaginal discs, and E2F1 is specifically turned over during early S-phase in embryos and larvae (Shibutani *et al.* 2007; Shibutani *et al.* 2008; Acharya *et al.* 2010).

RB proteins also are required in tissue- and stage-specific manners, as seen in studies of different metazoan RB family members. Although expressed, the *Drosophila* Rbf1 protein is not functionally required for early cell-cycle regulation in the embryo (Du and Dyson 1999; Stevaux *et al.* 2002; Keller *et al.* 2005). In the mouse, early embryonic requirements for RB are restricted to the trophectoderm, although the protein is expressed in other tissues (Wu *et al.* 2003). The *Caenorhabditis elegans lin-35* (RB homolog) mutant shows a largely nonoverlapping set of genes that are misregulated in embryo, L1 and L4 larvae (Kirienko and Fay 2007). These and other studies emphasize that the multifarious functions of this protein family will require global studies in a developmental setting. A major objective along these lines is the identification of functional and physical target genes of RB family corepressors.

Among the best-characterized targets of RB family proteins are genes such as *PCNA* and *DNA pol alpha*, which are involved in cell-cycle regulation; however, RB proteins also regulate a variety of genes involved in other pathways, such as apoptosis, DNA repair, and differentiation (Lipinski and Jacks 1999; Classon and Harlow 2002; Kirienko and Fay 2007). In *Drosophila*, functional targets of Rbf and E2F proteins were identified by transcriptomic analysis of *Drosophila* S2 cells (Dimova *et al.* 2003). Physical and functional targets of the MMB/dREAM complex, with which Rbf1 is also associated, were identified in *Drosophila* Kc cells (Georlette *et al.* 2007). These studies suggest that Rbf1, and to a lesser extent Rbf2, interact with distinct classes of genes that show varying sensitivity to loss of Rbf and E2F proteins. Targets of mammalian RB, p107, and p130 proteins were identified by chromatin immunoprecipitation (ChIP) in human lung fibroblasts; the proteins are observed to be redeployed in response to expression of the RB-binding adenoviral E1A protein (Ferrari *et al.* 2008). The genome-wide occupancy of RB and p130 also was reported recently in growing, quiescent, and senescent human fibroblasts, indicating that these proteins bind to thousands of putative target genes (Chicas *et al.* 2010). However, until now no study has presented a picture of the genome-wide occupancy of RB proteins in a whole organism during development. Using highly specific antibodies developed against the endogenous Rbf1 protein, we carried out ChIP to study Rbf1 protein occupancy through developmental time and used parallel sequencing (ChIP-seq) to identify genome-wide targets of Rbf1 in the *Drosophila* embryo. These results identify a diversity of potential Rbf1 targets and promoter composition and suggest that in addition to known links to cell cycle, this protein may play a direct role in the control of numerous conserved signaling pathways that are linked to metabolic regulation and growth.

## Materials and Methods

### Fly stocks

Embryos of a *Drosophila melanogaster yw^67^* strain were used for all ChIP assays.

### Reporter constructs and luciferase assay

To further analyze target genes bound by Rbf1, upstream promoter regions of *InR* from −1000 to −1, *Mer* from −600 to +400, *Rab23* from −900 to +100, *Hpo* from −600 to +61, *Dad* from −500 to +100, *p53-proximal* from −204 to +50, *Stat92E* from −500 to +152, and *Act5C* from −900 to +100 with respect to the transcriptional initiation sites were PCR amplified and cloned into *Xho*I and *Asc*I sites in pAC2T-luciferase vector (Ryu and Arnosti 2003). Each clone contained the portion of DNA bound by Rbf1 in the embryo. In addition, the *PCNA*-luciferase reporter was used as a positive control (Acharya *et al.* 2010). *Drosophila* S2 cells were transfected using Effectene transfection reagent (QIAGEN, Valencia, CA) according to the manufacturer’s protocol. A total of 1.5 million cells were transfected with 600 ng of one of the luciferase reporters, 250 ng of pRL-CMV Renilla luciferase reporter (Promega, Madison, WI), and 250 ng of pAX-*rbf1* (Acharya *et al.* 2010), or 20 ng of pIE4-*myc-E2F1* (Frolov *et al.* 2001). Cells were harvested 72 hr after transfection, and luciferase activity was measured using Dual-Glo Luciferase assay system (Promega) and quantified using the Veritas microplate luminometer (Turner Biosystems, Sunnyvale, CA).

### Chromatin immunoprecipitation

**Figure 1:**
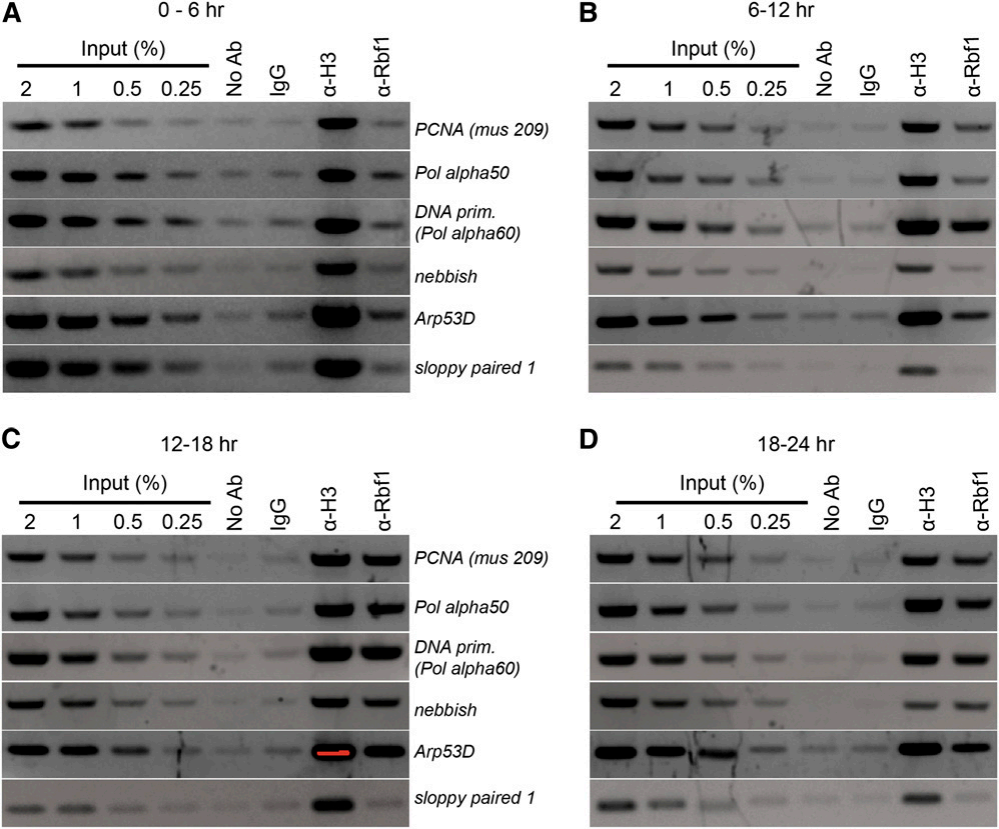
Rbf1 exhibits dynamic promoter occupancy. Rbf1 occupancy of regulated promoters measured by ChIP was low in 0- to 6-hr embryos and peaked at 12 to 18 hr. Formaldehyde cross-linked chromatin was prepared from embryos of different ages and immunoprecipitated using the indicated antibodies. No specific enrichment was found at a nontarget gene promoter (*sloppy paired 1*). No Ab indicates immunoprecipitation carried out without antibody; IgG, nonspecific mouse polyclonal antibodies; α-H3, antihistone H3 antibody; and α-Rbf1, rabbit anti-Rbf1 antibody.

Chromatin immunoprecipitations were conducted using *yw Drosophila melanogaster* embryos collected at room temperature and aged as indicated in Figure 1. For supporting information, Figure S3, embryos were collected from *Drosophila melanogaster* harboring Flag epitope tagged *rbf1* (Acharya *et al.* 2010). Fixing and chromatin preparation was carried out as described in File S1; immunoprecipitations were carried out overnight at 4° using polyclonal rabbit anti-Rbf1 serum (Keller *et al.* 2005). The Rbf1 antibody does not crossreact with Rbf2 on western blots, and it detects only the appropriately sized polypeptide in a western blot of whole embryo extracts (Keller *et al.* 2005).

### Sequencing of immunoprecipitated DNA fragments

The double-stranded DNA ends were repaired with T4 DNA polymerase, Klenow fragment, and T4 PNK enzymes. After a second purification step, an adenine-residue was added with Klenow [3′>5′ exo^−^] enzyme and again purified on Quiaquick columns. Adapters from Illumina for LM-PCR were then ligated to the end of the DNA molecules. The product of the reaction was then run on a 2% NuSieve agarose gel, and a band corresponding to 200 bp was extracted and purified. A total of 20 cycles of PCR were performed using Phusion polymerase (Finnzyme F-530S) and the Illumina oligos, and the products were purified by gel electrophoresis. High-throughput sequencing was performed on an Illumina Genome Analyzer with standard Illumina 36 cycles reaction kit. The DNA libraries generated (two Rbf1 and one preimmune) were sequenced in one lane each.

### Mapping the reads, peak finding, and visualization

We obtained 13,909,250, 18,070,094, and 1,247,796 reads for two anti-Rbf1 immunoprecipitation libraries and one control library, respectively. The quality-filtered 36-bp short sequence reads were aligned to *Drosophila melanogaster* genome (Flybase build r5.28) using Bowtie 0.12.3 (Langmead *et al.* 2009) with default parameters except that sequences were required to map uniquely to the genome (setting –m 1). To identify Rbf1 bound regions, QuEST software (Valouev *et al.* 2008) version 2.4 was used with relaxed stringency. The peaks were visualized using the online version of UCSC genome browser. We identified 2187 and 1337 peaks in two biologic anti-Rbf1 immunoprecipitation replicates; there were 1236 peaks that were present in both replicates, which we termed Class A peaks. An additional 951 and 101 nonoverlapping peaks are termed Class B and Class C peaks respectively (Table S1). Class A peaks were used for the all analyses except for the signaling pathway components (Figure 3) where Classes A, B, and C peaks were used. Intensities of peaks in first replicate were greater (from 10 to 656) than in the second replicate (from 10 to 109). The intensities were on average greater for Class A peaks than for Class B, and greater for Class B than C. We list the greater intensities obtained from the first replicate experiment in Table S1 for Class A peaks.

### Validation of ChIP-seq peaks

To independently assess enrichment of Rbf1 on novel target genes, several genes were selected and their enrichment in ChIPed chromatin was tested by PCR (Figure S1). The immunoprecipitated material that was also used for Illumina sequencing was used to validate the ChIP-seq peaks. The oligonucleotides used for PCR are listed in Table S6.

### Determination of peak overlap in replicates, *de novo* motif discovery, and motif analysis

Peaks observed in the two Rbf1-immunoprecipitate experiments for which the maximum points were located within 200 bp of each other were considered to be overlapping peaks. For each peak region, 100-bp sequences on each side of the peak maximum height location were extracted using a Perl script (output_genomic_regions_from_calls.pl) that was obtained from QuEST website (http://www-hsc.usc.edu/~valouev/QuEST/QuEST.html). Motif discovery was performed using MEME suite version 4.3.0 (Bailey and Elkan 1994). The program was set to search for overrepresented 5 through 15 mer motifs separately. The top four overrepresented motifs identified for each k-mer were selected and compared against TRANSFAC and JASPAR databases using the online version of TOMTOM (http://meme.ebi.edu.au/meme/intro.html). In most cases, similar motifs were found for different lengths (5−15 mers); the motifs shown in Figure 6A represent the shortest version of the motifs found to be overrepresented. We determined the quality of individual motifs compared with the defined consensus sequences using MAST (MEME suite version 4.3.0) on the sequences extracted from peak regions. To determine the significance threshold that would provide the best discrimination between enriched motifs and chance sequences shown in Figure 6B, the sequences under the peaks were randomized five times and MAST was run for each scramble independently. A *P* < 0.0001 was found to provide the greatest difference between the percentage of randomized promoters containing the overrepresented motif and the percentage of Rbf1-bound regions. To determine whether the overrepresented motifs identified were enriched specifically on promoter regions associated with Rbf1 binding shown in Figure 6C, DNA sequences extending from −100 to −300 from 1000 randomly selected Drosophila promoters not bound by Rbf1 were used for background analysis using MAST, and this process was repeated for total of five different sets of one thousand non Rbf1-binding promoters.

### Gene ontology (GO) analysis

Genes with transcriptional start sites (TSS) within 2 kb from peak maximum were considered associated with the peak. A total of 1169 of 1236 Class A peaks were mapped to TSS of 1890 genes. The enrichment of gene ontology terms was performed using online tool DAVID (Huang da *et al.* 2009) and 321 original GO categories were identified. Using visual inspection, related GO terms such as cell cycle, mitotic cell cycle, and meiotic cell cycle were pooled into one broader category, Cell Cycle and DNA replication. In this way, the GO terms with significant enrichment were pooled into a total of 10 different categories. Other terms with fewer genes and less significant enrichment were grouped under the “others” category. We report in Figure 5 the *P* values for the most populous subcategory of each super category shown on the pie chart.

### Identification of human orthologs to genes bound in *Drosophila* by Rbf1

The human orthologs of the 1890 genes associated with Class A peaks of Rbf1 in *Drosophila* were obtained from FLIGHT (http://flight.icr.ac.uk/). To determine conservation of RB family binding to conserved signaling pathway genes, the 295 genes listed on Table S8 were input into FLIGHT. An ortholog was considered bound in both fly and human if at least one member of an orthologous family was occupied in each species.

## Results

### Rbf1 exhibits developmentally regulated promoter occupancy

In previous studies, we and others showed that Rbf1 protein is expressed throughout embryogenesis, although the cofactor is not required for early cell cycles (Du and Dyson 1999; Stevaux *et al.* 2002; Keller *et al.* 2005). To investigate possible temporal control of Rbf1 binding to target gene promoters, we performed ChIP using 0- to 6-, 6- to 12-, 12- to 18-, and 18- to 24–hr-old embryos. Enrichment of Rbf1 protein was studied at selected promoters from different classes of Rbf1-responsive genes, described previously (Dimova *et al.* 2003). In all cases, Rbf1 occupancy was low in early embryos with a peak in 12- to 18-hr embryos (Figure 1 and Figure S1). These results indicate that Rbf1 promoter association is developmentally regulated; the lack of early Rbf1 association coincides with the rapid early cell cycles that lack G1 and G2 phases.

### Characterization of genome-wide Rbf1 association

Although many well-characterized targets of RB family proteins are genes involved in cell-cycle regulation and DNA replication, genes involved in other processes are also functionally regulated by these corepressors (Classon and Harlow 2002; Dimova *et al.* 2003; Kirienko and Fay 2007). To develop a global understanding of the genomic targets Rbf1, we used ChIP-seq technology. We prepared chromatin from 12- to 18-hr embryos where robust signals had been detected by conventional ChIP (Figure 1 and Figure S1) and generated separate DNA libraries from two Rbf1 immunoprecipitation experiments and one experiment using preimmune antibodies. The anti-Rbf1 libraries yielded ~14 and 18 million reads, whereas the preimmune serum library generated considerably fewer reads (1.2 million). Approximately 60% of the reads were uniquely alignable to the *Drosophila melanogaster* genome; other reads were found to map to more than one site or did not align at all using the strict criteria employed. A total of 1236 peaks were found in both anti-Rbf1 immunoprecipitations, whereas an additional ~1000 peaks were also found, mainly in one of the two ChIP experiments that exhibited more robust peak intensities. The preimmune control ChIP results showed an even distribution of very low peaks as would be expected from nonspecifically precipitated material (data not shown). In contrast, specific signals were reproducibly observed for both ChIP-seq samples. The overall low background found with the preimmune immunoprecipitation and reproducibility between the two biological replicates provides a high level of confidence for many of the peaks. Of the 1236 high-confidence peaks recovered in both of the biological replicate experiments, approximately 95% could be mapped to within 2 kb of predicted transcriptional start sites of known genes. These peaks were proximal to transcriptional start sites of 1890 genes. Additional strong candidate genes were found in the library that yielded 18 million reads, generating a total of 3188 genes.

### Rbf1 target genes indicate regulation of RB pathway at multiple levels

Clear signals were observed on a number of promoters that we expected to find in this data set, including *DNA pol alpha*, *DNA primase*, and *PCNA*, known physical targets of Rbf1 (Stevaux *et al.* 2002; Dimova *et al.* 2003). To validate the process of Illumina sequencing and peak calling used to generate this data, we selected a set of promoters to independently analyze by direct PCR; positive and negative signals were confirmed in all cases (Figure S2 and data not shown). We also carried out experiments with a different antibody to test the reproducibility of these ChIP results. Chromatin was prepared from flies harboring transgenic Flag-epitope tagged Rbf1 (Acharya *et al.* 2010), and immunoprecipitations were carried out using either anti-Rbf1 or Flag antibodies (Figure S3). In each case, ChIP signals coincided exactly.

**Figure 2:**
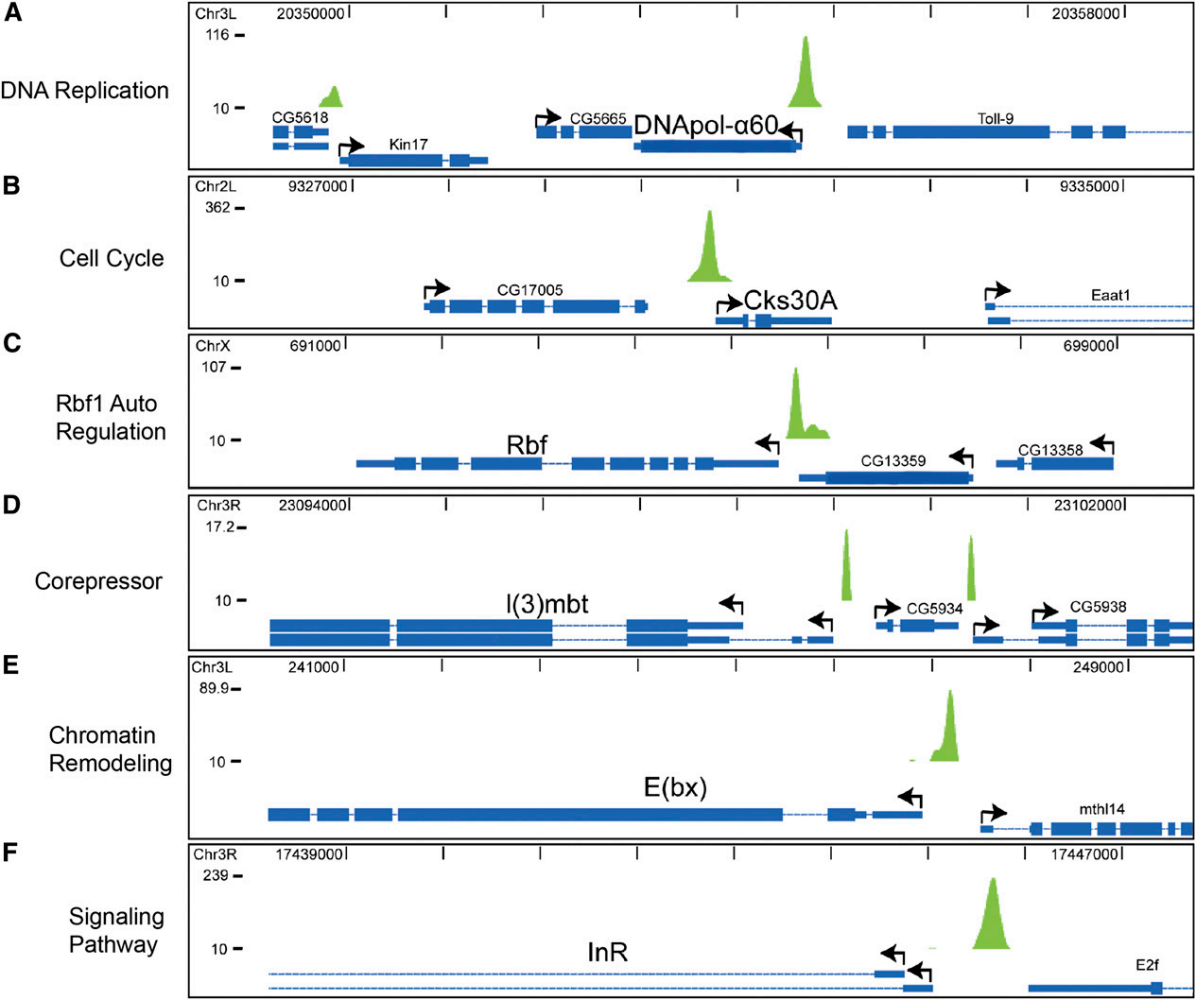
Rbf1 promoter-proximal occupancy of diverse classes of genes suggests autoregulatory effects. Strong peaks were noted on cell-cycle related genes, such as *DNApol-α60* and *cyclin-dependent kinase 30A* (A, B). Autoregulation is suggested by occupancy of the *Rbf* gene (C). The 5′ region of the Rbf1-related corepressor *l(3)mbt* is also associated with Rbf1 (D). The promoter of the *dNURF 301/E(bx)* gene, a chromatin remodeling component important for RB function in development, is also bound (E). Numerous components of cell signaling pathways, including the insulin receptor *InR*, also are targeted by Rbf1 (F). Relative peak intensities are shown on the Y-axis. Representative individual peaks are visualized on the UCSC genome browser. Bent arrows indicate the direction of transcription of the genes and absence of arrows indicates 3′ region of a gene.

We found that diverse classes of genes were targeted by Rbf1, including a set of genes that indicates that Rbf1 may regulate its own functional output at multiple levels (Figure 2 and Table S1). The autoregulatory properties of Rbf1 were suggested by particular genes, such as *cyclin A*, *B3*, *E*, and *cdk4/6*, which encode the kinase complexes that downregulate Rbf activity; the *rbf1* gene itself; and *cyclin-dependent kinase subunit 30A*, a component of the Cdk1−cyclin B kinase complex that phosphorylates numerous proteins involved in DNA replication, translation, and chromatin structure (Holt *et al.* 2009). Consistent with this notion, negative feedback loops of regulation of *RB* and *p107* have been reported in mammalian cells (Burkhart *et al.* 2010a,b). A peak was also associated with the 5′ promoter of the *l(3)mbt* gene, whose protein product is a member of a conserved MMB/dREAM transcriptional regulatory complex that also involves the Rbf1 protein (Lewis *et al.* 2004).

Other MMB/dREAM components were also targeted by Rbf1, consistent with autoregulation of the entire complex by Rbf1 and MMB/dREAM (Lewis *et al.* 2004; Tabuchi *et al.* 2011). Rbf1 peaks also were associated with additional chromatin-regulatory components, including the *E(bx)* gene, which encodes the NURF301 component of a SWI/SNF nucleosome remodeling complex that has been shown to antagonize the action of RB in *C. elegans* development (Andersen *et al.* 2006). These data indicate that Rbf1’s direct transcriptional regulation may well control RB pathway output at five levels; Rbf activity via cyclins and kinases that directly phosphorylate the protein, production of *rbf1* transcripts, of cofactors that work together with Rbf proteins, of factors that antagonize Rbf activity, and kinases that are implicated in cell-cycle regulation of downstream genes. The potential effect of this regulatory structure is that changes in Rbf protein abundance or activity will reset levels of other components that would magnify or dampen the control of the entire Rbf regulon.

### Rbf1 target genes include multiple components of conserved signaling pathways

**Figure 3:**
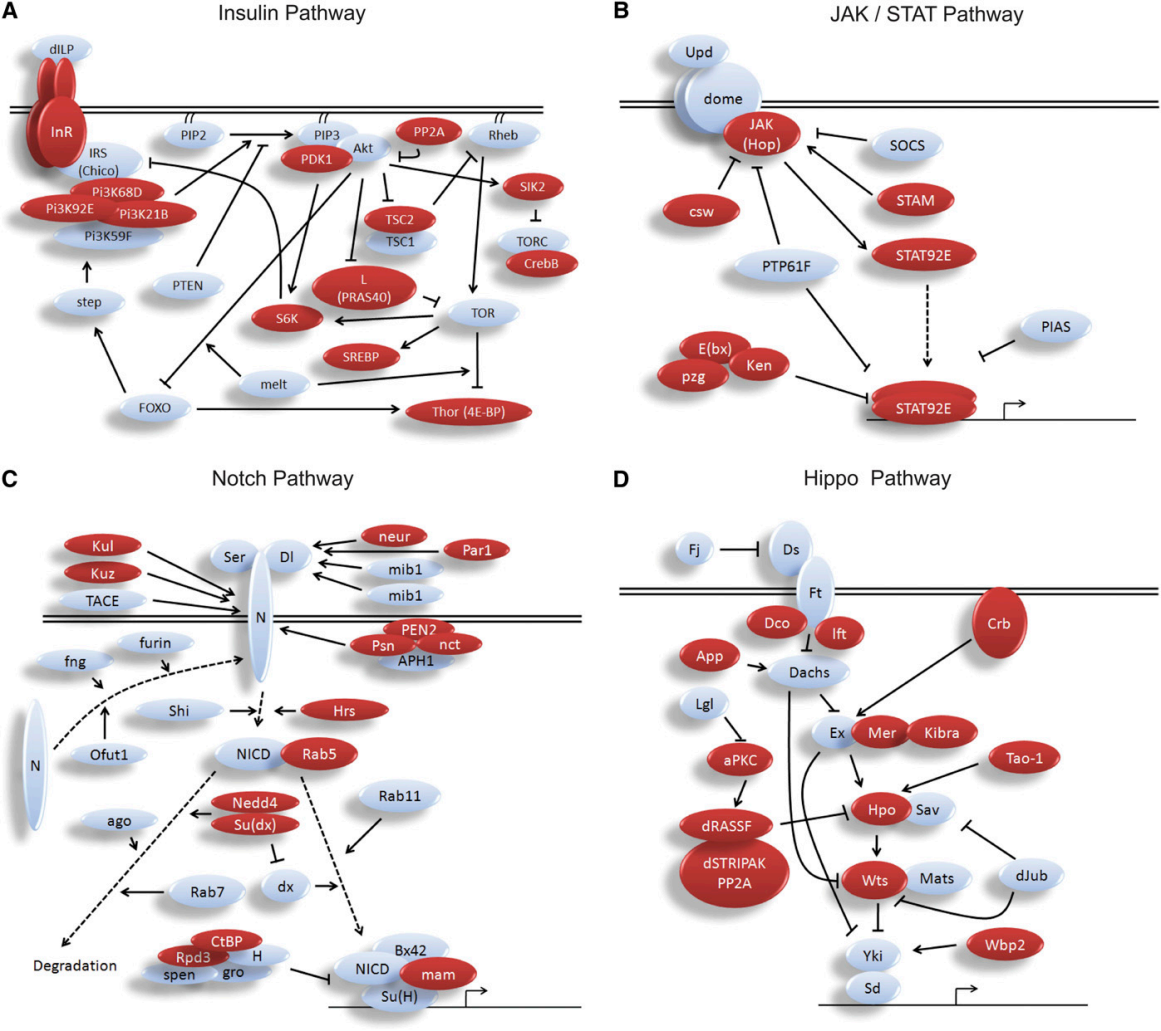
Rbf1 occupies multiple nodes in conserved signaling pathways. Genes in the insulin (A), JAK/STAT (B), Notch (C), and Hippo/Warts/Yorkie (D) signaling pathways are targeted at multiple levels by Rbf1. Proteins of genes targeted by Rbf1 are indicated in red.

One of the most striking observations about the roster of genes occupied by Rbf1 was its extensive and hitherto unappreciated occupancy of genes involved in essential, conserved signaling pathways. One of the strongest Rbf1 peaks is found proximal to the insulin receptor promoter (Figure 2F). Further investigation of the 3188 gene data set revealed that Rbf1 peaks were extensively associated with other components of insulin signaling, including three of the four *Drosophila* PI3 kinase genes, S6 kinase, and Thor/4E-BP (Figure 3 and Table S2). JAK/STAT signaling components identified as Rbf1 targets include the signal mediator JAK kinase, the STAT92E transcriptional effector, as well as regulators of this pathway, Ken, E(bx), Pzg, and STAM. A large number of the genes in the Hippo growth-control pathway, including those for the central Hippo and Warts kinases, are bound by Rbf1. Components of the Notch signaling pathway, in particular regulatory proteases that process the Notch protein, were also found among Rbf1 target genes. Wingless, Hedgehog, nuclear factor-κB, transforming growth factor-β, target of rapamycin, epidermal growth factor receptor/Ras, and JNK pathway components also were bound by Rbf1. In total, we identified 137 signaling pathway genes that were bound by Rbf1; 75 of these were clearly identified in both ChIP-seq samples, with a further 13 present as peaks in both datasets but just below the cutoff in one of the ChIPs.

Association of Rbf1 with signal pathway genes was confirmed in additional biological ChIP experiments (Figure S2, Figure S3, and data not shown). The promoter-proximal positions of most Rbf1 peaks, as well as the comparatively small number of regions bound by Rbf1 overall, suggest that these binding events are not simply the effect of large numbers of promiscuously bound Rbf1 proteins. Genes for almost one-half of insulin signaling and more than one-half of Hippo signaling components were occupied by the Rbf1 protein compared with the genome-wide average of 22%, indicating a strong enrichment. The extensive physical interaction between Rbf1 and components of diverse signaling pathways suggests a novel means by which cell-cycle information may be integrated with the information related to metabolic status, organ and tissue size, and differentiation states. The occupancy of genes located at multiple levels of signaling, such as the insulin receptor and the S6 kinase genes, indicate that Rbf1 may be in a position to regulate these pathways in a complex, multifactorial manner.

To test whether Rbf1 indeed represses some of these novel targets, we created *InR* (insulin signaling) and *Rab23* (hedgehog signaling) reporter constructs and measured their sensitivity to Rbf1 overexpression compared with *Act5C* and *PCNA* controls. As expected, Rbf1 effectively repressed the *PCNA* reporter and not the *Act5C* reporter. Significantly, strong inhibition of *InR* and *Rab23* promoters was observed, indicating that Rbf1 can functionally regulate in S2 cells at least some of the novel targets identified (Figure S4). Removal of the Rbf1 binding region from an *InR* reporter inhibited this repression by Rbf1 (Y. Wei, unpublished data). Our study clearly indicates potential involvement of Rbf1 in regulating signaling pathway components; however, the functional significance of these physical interactions remain to be investigated, and further research will be required to fully understand possible crosstalk between Rbf1 and conserved signaling pathways.

### Rbf1 exhibits a strong promoter-proximal targeting bias

**Figure 4:**
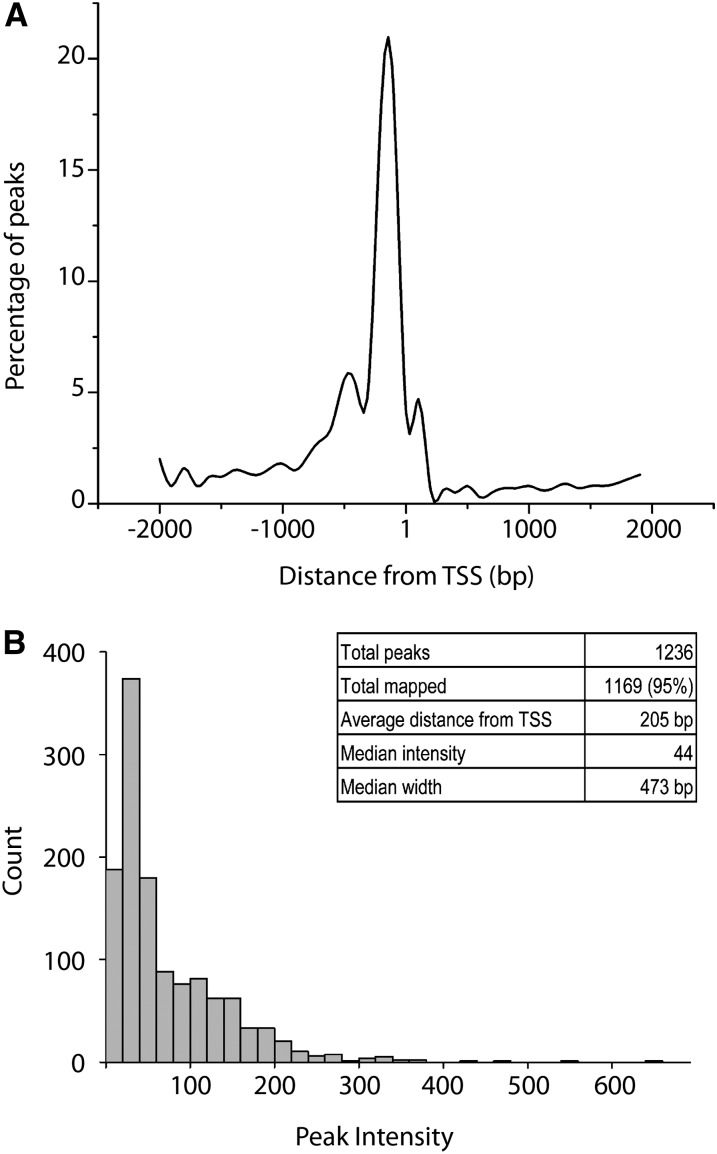
Rbf1 exhibits a strong promoter-proximal targeting bias. (A) The distribution of peaks relative to the nearest TSS. The majority of peaks are centered 205 bp 5′ of the TSS. Distances were grouped into 100-bp bins and points fitted with a smooth curve. (B) Distribution of peak intensities. Most peaks had an intensity within a few fold of the average, although some peaks were >10-fold greater.

As noted previously, most Rbf1 peaks were located within 2 kb of transcriptional initiation sites; mapped on a finer scale, we found that there was a strong preference for binding centered at −205 bp (Figure 4). Unlike other corepressors such as CtBP and Groucho, the strong preference of Rbf1 for the 5′ ends of genes suggests that Rbf1 can only exhibit activity near the initiation site, or that transcription factors that it interacts with, such as E2F proteins, can only activate effectively from promoter-proximal locations. Interestingly, Rbf1-associated MMB/dREAM complex proteins are also found to bind in promoter-proximal locations; neither this complex nor Rbf1 alone appear to frequently interact with distal *cis*-regulatory sequences (Georlette *et al.* 2007). Rbf1 peak intensities (representing the number of sequences recovered for particular genomic positions) spanned approximately an order of magnitude, with relatively few outliers showing more than tenfold greater than the median value. Overall peak heights were not correlated to the types of genes targeted, as genes in functionally related classes exhibited peaks of a range of intensities. As discussed below, the sequences underneath the peaks presented a very heterogeneous picture, indicating that different transcription factors may provide alternative pathways for recruiting Rbf1.

### Cell-cycle and DNA replication−related genes represent only a minority of bound sites

**Figure 5:**
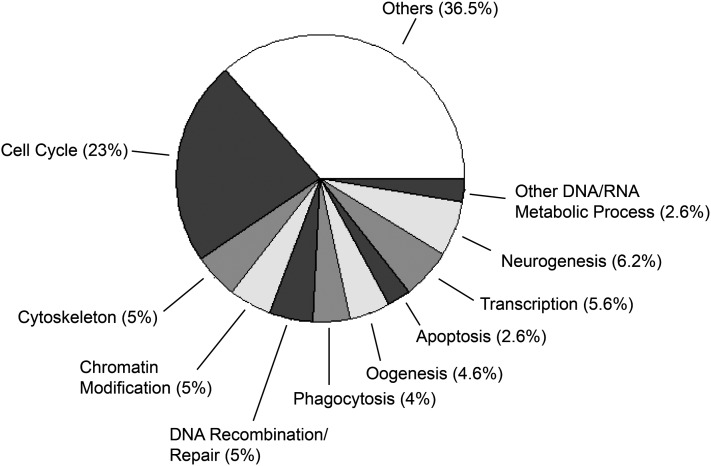
Rbf1 target genes represent diverse GO categories. A total of 42% of 1890 Rbf1 target genes were enriched for GO terms. Of these, only approximately one-quarter were associated with Cell Cycle and DNA replication, whereas the majority of targets grouped into other gene regulatory and developmental processes. The GO terms are arranged in the pie chart in decreasing order of significance of enrichment from Cell Cycle and DNA replication to Other DNA/RNA Metabolic Process. “Others” indicates numerous smaller groups of enriched genes. The *P* values for the categories are as follows: Cell Cycle, 7E-16, Cytoskeleton, 5E-13; Chromatin Modification, 2E-12, Recombination/Repair, 7E-09; Phagocytosis, 1E-03; Apoptosis, 5E-03; Transcription, 2E-02; Neurogenesis, 2E-02; and other DNA/RNA Metabolic Process, 4E-02.

The previous functional assessment of genes regulated by Rbf in cell culture had highlighted genes related to cell cycle and DNA replication, although a much broader spectrum of gene functionalities was indicated by analysis of genes regulated and bound by the Rbf-related MMB/dREAM complex (Dimova *et al.* 2003; Georlette *et al.* 2007). To characterize the nature of Rbf1 direct targets, we performed GO analysis on genes associated with the greatest confidence peaks using the DAVID annotation analysis system (Huang da *et al.* 2009). Of 1890 Rbf1 target genes, 42% were enriched for GO terms. Rbf1 peaks were associated primarily with protein-coding genes, but 12 annotated noncoding RNA genes also were associated with the cofactor. Approximately one quarter of the genes were enriched for cell cycle and DNA replication categories, the areas that showed the most significant enrichment of all groups of genes (Figure 5 and Table S3). Other categories that were enriched included processes, such as chromatin modification and transcription, cellular systems, such as cytoskeleton, and developmental programs, including oogenesis and neurogenesis. A large number of smaller categories comprised 36% of the target genes. Previous studies have shown that RB family members have particular roles in distinct developmental settings, such as the role for RB in mouse trophectoderm development, vulval development in *C. elegans*, and osteoblast differentiation (Wu *et al.* 2003; Bender *et al.* 2007; Berman *et al.* 2008). The smaller number of noncell-cycle–related genes previously found to be functionally regulated by Rbf1, Rbf2 and E2F proteins indicate that perhaps in cell culture, many physiological targets of these proteins are relatively quiescent (Dimova *et al.* 2003). Our data suggest that taken in a developmental context, Rbf1 function may be distributed over a very wide set of diverse cellular processes.

### Enrichment of transcription factor motifs in Rbf1-bound peaks

**Figure 6:**
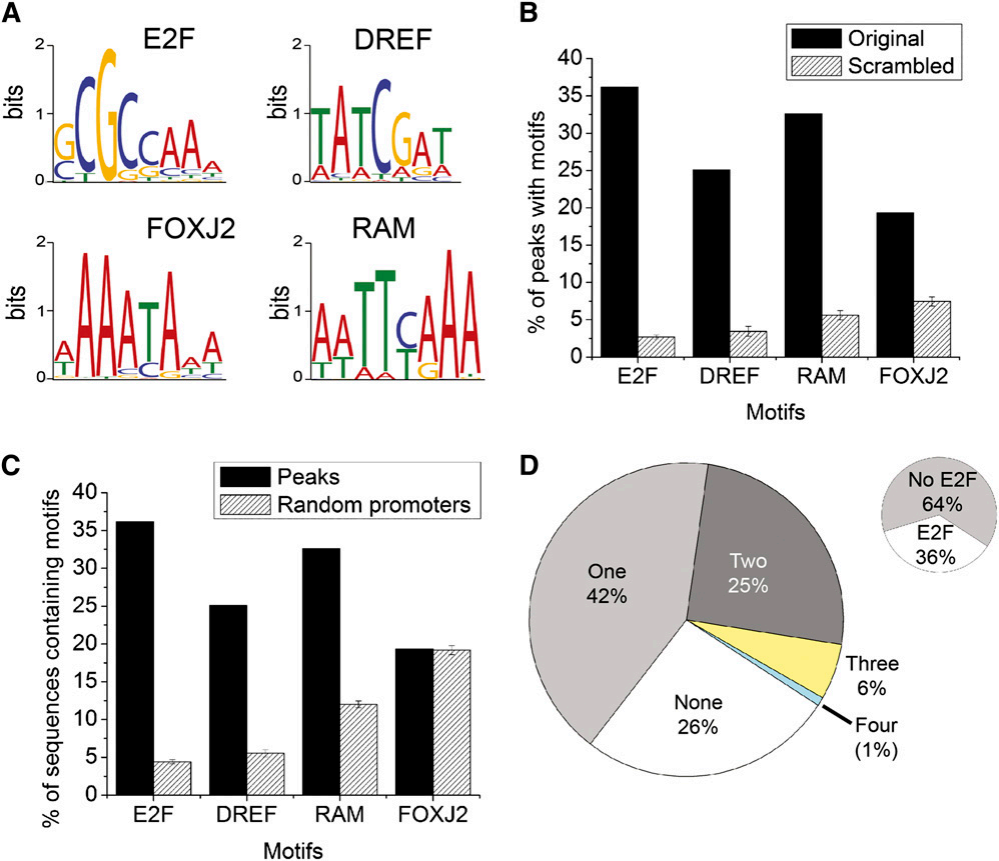
Transcription factor motifs enriched in Rbf1-bound peaks. (A) The four most overrepresented motifs identified by the MEME motif discovery tool, including one previously unknown motif (RAM). (B) Rbf1-associated motifs are highly enriched compared with average occurrence in DNA of the same A/T composition. The sequences under Rbf1 peaks were scrambled five times, and specific motifs with *P* < 0.0001 were identified. E2F sites showed the greatest level of enrichment in specifically bound regions compared with randomized DNA sequences. (C) E2F, DREF, and RAM motifs preferentially associate with Rbf1-bound promoters. The presence of motifs in Rbf1-bound sequences was compared with the presence in Rbf1-unbound promoters. FOXJ2 sites are not restricted to Rbf1-associated promoters and may represent a motif for a broadly acting factor. Note that the canonical DREF sites are 8-mers (Yamaguchi *et al.* 1995). In our data, the eighth nucleotide was not conserved. (D) Diversity of motif composition of peaks. A total of 42% of total peaks contained only one of the four different motifs (E2F, DREF, FOXJ2, or RAM). A quarter of the peaks had a combination of two different motifs; 6%, a combination of three, and 1% contained all four motifs. Only 36% or peaks had an identifiable E2F motif (small insert). Strikingly, a quarter of the peaks did not have any of the four motifs. The heterogeneity of sequences in Rbf1-bound peaks suggests that E2F may not be the only transcription factor that recruits Rbf1 to target gene promoters. Peaks used in this analysis were drawn from the 1236 bound regions found in both Rbf1 ChIP biological experiments. A peak with multiple E2F motifs, but no other motif types, was counted as one type of motif; a similar treatment applies for the other three motifs. “None” means the peaks did not contain any motifs for E2F, DREF, FOXJ2, or RAM.

Rbf1 does not bind to DNA directly but is instead recruited by transcription factors, generally of the E2F family. RB has been reported to interact with other types of transcription factors, including MyoD, NeuroD1, GATA1, and components of the RNA polymerase III basal transcriptional machinery (Gu *et al.* 1993; Felton-Edkins and White 2002; Batsche *et al.* 2005; Kadri *et al.* 2009). In consideration of the wide diversity of genes targeted by Rbf1, we sought to understand whether particular subsets of these genes would be characterized by distinct constellations of protein binding sites in the promoter proximal regions. We extracted sequences representing the 200-bp sequences from the center of each peak and performed *de novo* motif discovery analysis using MEME (Bailey and Elkan 1994). We sought the top five overrepresented motifs for 5 to 15 mers and noted that variations of four motifs occurred most frequently (Figure 6A). We searched the motifs in the JASPAR and TRANSFAC databases, and one of the motifs was similar to the E2F site, as expected. Another bears clear similarity to the DREF site, which is bound by the DNA replication-related element-binding factor, involved in the expression of a wide variety of proliferation-related genes and whose function in the context of cell-cycle regulation has been investigated on the *PCNA* and *DNApol alpha* promoters (Yamaguchi *et al.* 1995; Takahashi *et al.* 1996; Seto *et al.* 2006). We also found a motif similar to the binding sequence of the mammalian Forkhead transcription factor FOXJ2, and one novel motif that did not closely match any other sequence in the database, which we designated RAM (Rbf associated motif). The motifs occur much more frequently than they would in scrambled sequences of similar composition, indicating that these motifs have a high information value (Figure 6B). The threshold for calling each of the motifs (*P* = 0.0001) was selected to provide a high discrimination between the bound sequences and scrambled DNA of similar composition (Figure S5). Promoters selected from those in the genome that did not exhibit Rbf1 binding were tested for frequency of these motifs; E2F, DREF, and RAM motifs were considerably less enriched on these promoters than on those bound by Rbf1 (Table S4), indicating that these sequences may play a role in recruiting of Rbf1, or coregulation of the associated promoters (Figure 6C). FOXJ2 sequences were not preferentially enriched on Rbf1 bound promoters, although these sequences occur at a higher frequency than would be expected by chance, thus it is likely that these motifs are relevant to promoter function in general.

Regarding overall promoter composition, just less than one-half of the peaks contained at least one copy of one of the four motifs identified. A quarter of the peaks had a mixture of two of the motifs, and a small percentage had three or all four of the motifs (Figure 6D). One-fourth lacked any of these motifs; these promoters may contain novel motifs that are not found in many genes, or they may contain low-affinity canonical sites that fell below the threshold used here (see *Materials and Methods*). Interestingly, of the peaks that contained recognizable motifs, only about a third contained the E2F motif, although this has been presumed to be the chief route by which Rbf proteins are recruited to promoters (Figure 6D insert). There may be other factors involved in recruiting Rbf1, or low-affinity E2F sites may be important on some genes. Indeed, Rbf1-bound regions as a group tend to be enriched in E2F-like sites (Figure S6). Although this manuscript was under review, a similar study from the Dyson laboratory (Korenjak *et al.* 2012) showed that in larvae, Rbf1 is recruited to some promoters that lack canonical E2F sites by E2F2, and that binding is abolished in a *dp* mutant background. This evidence strongly suggests that Rbf1 association is E2F dependent, whether or not recognizable E2F sites are present. On the basis of the available data, a very large proportion of the genes identified in the larvae are also present in our dataset, suggesting that Rbf1 targets similar genes in these different developmental stages. Both studies combine chromatin from diverse tissues, thus there may be tissue-specificity in Rbf1 binding that remains to be elucidated.

### Enrichment of motifs in different promoter subclasses

**Figure 7:**
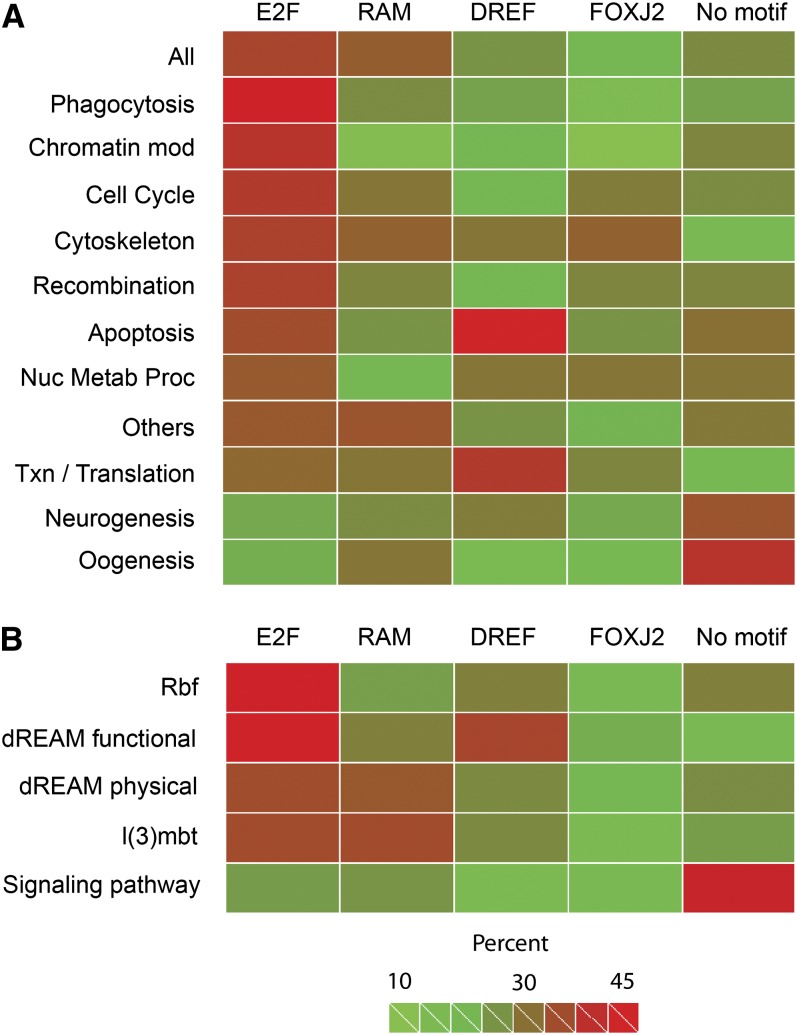
Enrichment of Rbf1-associated motifs indicates distinct promoter subclasses in the Rbf1 regulon. (A) Heat map for association of motifs with different GO categories. E2F sites were present in a significant fraction of bound regions as a whole, especially in GO categories Phagocytosis, Chromatin Modification, and Cell Cycle. Genes involved in Neurogenesis and Oogenesis tend to be depleted of E2F motifs. RAM sites tend to occur on a subset of E2F-containing sequences, but Chromatin Modification and Nucleotide Metabolic Process genes are depleted of RAM motifs. Apoptosis and Transcription/Translation genes are associated with DREF motifs. Chromatin Modification, Phagocytosis, and Oogenesis genes are depleted of FOXJ2 motifs, whereas a larger fraction of Neurogenesis and Oogenesis genes lack all of the four motifs. (B) Heat map for association of motifs with selected functional and physical targets of Rbf1, including functional targets of Rbf in S2 cells (Rbf); functional and physical targets of the Rbf- and Myb- containing dREAM complex; functional targets of l(3)mbt, a corepressor and a binding partner of Rbf; and physical targets of Rbf1 identified in this study that are involved in signaling pathways. The E2F motif alone is preferentially associated with Rbf1 functional targets in S2 cells (Rbf) and dREAM functional targets, however, a combination of E2F and RAM sites is found preferentially on l(3)mbt functional and dREAM physical targets. Signaling pathway Rbf1 target genes are depleted of all four motifs, suggesting a distinct promoter signature. “Percent” indicates fraction of genes in a selected GO category containing at least one occurrence of the indicated motif within the Rbf1 peak. The category “all” represents all 1236 peaks present in both ChIP replicates. The category “other” represents small clusters of genes found to be overrepresented in numerous GO categories (see Figure 5). “Signaling pathway” represents 136 genes found in diverse conserved *Drosophila* signaling networks (Table S3). Numerical values for heat map shown here are found in Table S5.

In light of the diverse cellular processes represented among the targets of Rbf1, we studied whether different classes of genes exhibited distinct promoter composition (Figure 7). Sorting genes by GO category, we noted that genes involved in Phagocytosis, Chromatin Modification, and Cell Cycle were among those most highly enriched in E2F motifs, whereas Neurogenesis and Oogenesis GO categories are depleted of these motifs. The novel RAM motif cooccurred frequently with E2F sites, except for certain GO categories such as Chromatin Modification while DREF sites, which had been previously shown to help regulate cell-cycle–related genes such as *PCNA* and *DNApol alpha*, were not overall enriched on this class of gene, instead showing strong association with genes involved in Apoptosis. FOXJ2 was also differentially distributed, showing some correlation with RAM sites, but it was not highly enriched in any subcategory (Figure 7A). The overall impression obtained from this analysis is that Rbf1-bound regions vary strongly in their average composition; it is likely that functional classes of genes are coordinately regulated by unique combinations of factors that interact with these motifs. Factors responsible for Rbf1 recruiting may vary as well, possibly placing some Rbf1-bound promoters out of reach of the canonical cell-cycle regulatory pathways.

We also analyzed the association of motifs with groups of genes drawn from our set of Rbf1-associated promoters that are defined by other properties, rather than the GO categories identified by DAVID. This analysis identifies a strikingly distinct signature for signal transduction genes that suggests Rbf1 may target these promoters through entirely different proteins. The separate categories analyzed included a set of genes affected by RNAi knockdown of Rbf1, Rbf2, E2F1, and E2F2 in S2 cells (Dimova *et al.* 2003), genes misexpressed by knockdown of the l(3)mbt malignant brain tumor protein that interacts with Rbf-containing complexes (Janic *et al.* 2010), functional targets of the MMB/dREAM complex identified by knockdown in Kc cells, physical targets of the Rbf1-associated MMB/dREAM complex components identified by ChIP (Georlette *et al.* 2007), and the set of genes that we identified in this study that are components of conserved signaling pathways (Figure 7B). Grouping genes in these five (nonexclusive) categories, we found that the E2F motif is especially enriched for functional targets of Rbf1 and MMB/dREAM identified in S2 cells. As noted previously, the motif is associated with cell-cycle–related genes, which would be expected to be expressed in these mitotically active cells. The E2F motif is also associated with phagocytosis-related genes, which may also be preferentially active in the S2 cell line, which has properties of hemocytes (insect macrophages). E2F sites were less enriched, and RAM sites were more enriched, on physical targets of MMB/dREAM and genes misexpressed in l(3)mbt brain tumor samples; these genes may represent developmentally regulated targets of Rbf1, as opposed to genes that are tightly integrated into regular mitotic control pathways (Lee *et al.* 2010). In the fifth group, genes involved in conserved signaling pathways, we noted that Rbf1-bound regions were depleted of all four motifs, suggesting a distinct promoter signature for these genes. Further bioinformatic analysis of that group as a whole did not find enrichment of new motifs, although weak E2F sites were found (data not shown).

E2F proteins have been observed to bind diverse sequences *in vivo* (Bieda *et al.* 2006). To directly test the possibility that some of the promoters with weak or nonexistent E2F sites may nonetheless interact with this transcription factor, we overexpressed E2F1 and measured its ability to activate a diverse panel of signaling pathway gene promoters. E2F1 strongly activated the *PCNA* promoter as expected, however, none of the signaling pathway gene promoters tested were activated by E2F1, indicating that these promoters are E2F-independent (Figure S7). Some promoters such as *InR* were slightly repressed, possibly because E2F1 may drive expression of the *rbf1* gene itself. Taken together, we see evidence that Rbf1 binding takes place in the context of a rich diversity of motifs among the different categories of genes targeted, suggesting that regulation of promoters by this corepressor may involve separate regulatory programs, consistent with recent studies (Dimova *et al.* 2003; Kirienko and Fay 2007; Lee *et al.* 2010).

### Divergence and conservation of Rbf1 regulon

**Figure 8:**
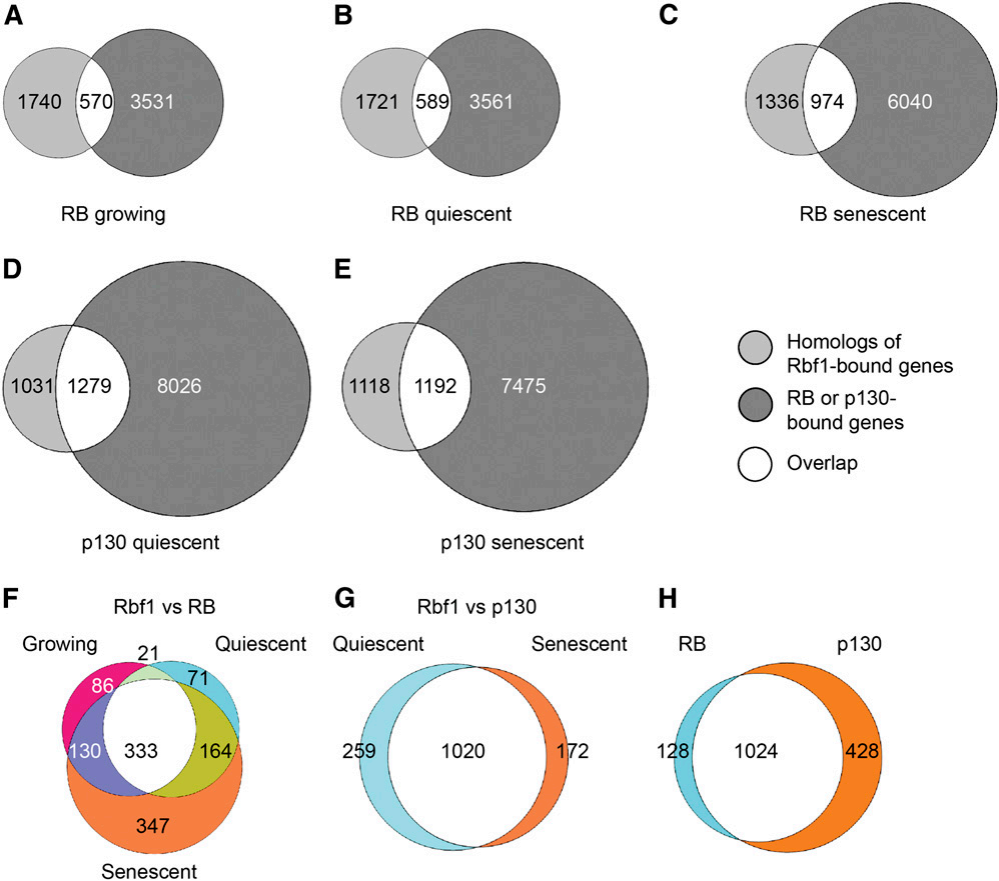
Divergence and conservation of RB regulon. A total of 2310 identifiable human orthologs of Rbf1 targets were compared with RB targets in growing (A), quiescent (B), and senescent (C) cells and p130 targets in quiescent (D) and senescent (E) cells. The overlaps in (A), (B), and (C) were further compared with each other (F) and the overlaps in (D) and (E) were compared with each other (G). Comparison of the total genes in (F) and (G) with each other (H), shows that most of the targets of Rbf1 and RB, as well as Rbf1 and p130, are the same. In all overlaps in A-E, GO terms DNA Replication, Cell Cycle, DNA Repair, and Chromatin Modification were enriched, indicating that these genes may form a conserved ancient regulon of RB proteins. The overlap of human homologs of Rbf1 targets in *Drosophila* embryos and RB and p130 targets in human cell culture suggests that many genes have retained regulation by RB proteins since divergence of these organisms. Other categories of genes may represent divergence of RB family function, or context-dependent differences in binding. Human homologs for Rbf1 targets were compared with published RB and p130 targets in growing, quiescent and senescent human lung fibroblasts (Chicas *et al.* 2010).

To determine whether the genomic targets of the *Drosophila* Rbf1 protein represent deeply conserved regulatory interactions, we compared human orthologs of the Rbf1-occupied genes with those bound by human RB and p130 proteins in fibroblasts (Chicas *et al.* 2010). The 1890 *Drosophila* genes identified in our study correspond to 2310 human orthologs. We compared these genes to those bound by RB or p130 in growing, quiescent, or senescent fibroblasts (Figure 8). Close to one-half of the orthologs were identified as RB targets under at least one condition, whereas just more than 60% of the orthologous genes were bound by p130 (Figure 8A-E). Among the genes bound by RB or p130, the GO categories DNA Replication, Cell Cycle, DNA Damage/Repair, and Chromatin Modification were under all conditions enriched. The GO category Cytoskeleton, which was found to be enriched in the Rbf1 targets in the *Drosophila* embryo, was actually depleted from genes bound by RB in growing and quiescent cells; however, it was enriched in genes bound by RB in senescent cells, as well as p130. This result indicates that certain categories of genes can be selectively occupied depending on the state of the cells, underscoring the differential regulation of subsets of RB/p130 targets. Finally, GO categories such as Oogenesis, Phagocytosis, and Neurogenesis that were overrepresented among *Drosophila* targets were slightly or not at all enriched in the set of human genes. The common binding of genes involved in chromatin modification, cell cycle, DNA repair and replication by RB family members suggests that they represent deeply conserved functions of this family of proteins. Other categories of genes may represent lineage-specific innovations or tissue-specific binding interactions that are not present in the cell culture system.

The intriguing targeting of many conserved signaling genes by Rbf1 in the *Drosophila* embryo led us examine whether RB/p130 show similar binding preferences. The vast majority (106/137) of signaling components bound in the fly were also found to have RB and/or p130 at the promoter in human fibroblasts. These corepressors were found at 111 additional signaling pathway genes that lacked Rbf1 occupancy in the embryo (Table S8). The high proportion of conserved signaling pathway genes targeted by RB family proteins suggests that this proposed regulatory connection may represent an essential link between multiple cellular components of growth control and differentiation.

## Discussion

Our analysis of genomic occupancy of Rbf1, the major RB protein in the *Drosophila* embryo, provides intriguing new pictures of activities of this conserved cofactor. Previous genetic studies of this factor showed that the protein is not required in early embryogenesis, despite the presence of this protein (Du and Dyson 1999; Stevaux *et al.* 2002; Keller *et al.* 2005). Consistent with this picture, our temporal analysis indicates that there is a widespread, perhaps universal, regulation of Rbf1 binding during this period of development, limiting access to promoter regions. Although phosphorylation of RB proteins is a well-studied pathway that regulates contact of the cofactor with E2F proteins, there is no evidence that RB proteins from early embryos show a preferentially hyperphosphorylated, slower-migrating form; thus, additional forms of regulation may be important for this developmentally controlled binding (Stevaux *et al.* 2002; Keller *et al.* 2005). In our analysis we found low levels of occupancy of target gene promoters by Rbf1 during early stages, especially 0- to 6-hr embryos. During this developmental time window, the nuclei in the embryo are either engaged in rapid mitotic cycles (cycles 1–13, where there are no G1 and G2 phases) or have just finished, and may not have yet established the regulated Rbf1-bound state. Further analysis of the occupancy of Rbf1-recruiting transcription factors during development will shed light on this matter.

Our study identified 1890 promoters that are bound by Rbf1; this is an order of magnitude higher than the number of genes identified as functional targets of Rbf/E2F factors in RNAi experiments conducted on cultured S2 cells (Dimova *et al.* 2003). A large majority of the genes misregulated in S2 cells after RNAi knockdown of Rbf1 were bound by the corepressor in the embryo, indicating that many of these genes are indeed direct Rbf1 targets, but the question remains how to interpret the other identified binding events. Some may represent fortuitous associations that do not materially contribute to gene regulation, as has been suggested for some genome-wide associations of transcription factors (Georlette *et al.* 2007; Li *et al.* 2008); however, the tight promoter localization puts the corepressor in a position that is very likely to influence basal promoter activity and indeed we show evidence that at least some of these promoters can be repressed by Rbf1. We favor the idea that only a fraction of the genes that we identified are affected in cell culture because S2 cells do not represent the complex mixture of differentiated tissues that we sampled in the embryo. Activation of some of these genes may require co-stimulatory signals that are lacking in the cell culture system. Alternatively, the RNAi knockdown may have not been extensive enough to uncover the true scope of Rbf1 regulation.

One of the most surprising findings of our study is the extensive occupancy of multiple nodes of conserved signaling pathways by Rbf1 (Figure 3 and Table S2). This aspect highlights one feature of RB biology that our ChIP-seq analysis in developing embryos has brought to the fore. Previous studies that examined the regulons or direct physical targets of RB proteins have not emphasized this striking aspect of the system (Dimova *et al.* 2003; Georlette *et al.* 2007; Kirienko and Fay 2007; Ferrari *et al.* 2008; Chicas *et al.* 2010), which may be partially due to the heavy reliance on cell culture systems for this information; certain promoters may only be bound in a developmental context. However, this aspect of RB biology may also have been overlooked in part because some GO categories do not specifically identify individual signaling pathway genes. We reanalyzed the genes identified as RB and p130 targets in human fibroblasts and found that more than 200 signaling pathway genes are bound in these cells (Chicas *et al.* 2010 and data not shown). An additional feature of the human cell data set is that RB and p130 in cultured cells appear to occupy a greater percentage of total promoters than does Rbf1 in the *Drosophila* embryo, which tends to obscure the enrichment of any particular set of genes.

There is abundant functional evidence linking RB and conserved signaling pathways. The Hippo growth control pathway has been recently found to control Rbf1 activity itself, suggesting that there are homeostatic feedback loops regulating Rbf and Hippo levels (Nicolay *et al.* 2011; Tschop *et al.* 2011). Previous functional studies linked RB regulation to individual components of the insulin signaling and S6 kinase pathways in mammals and plants (Hsieh *et al.* 2008; Annicotte *et al.* 2009; Mercader *et al.* 2009; Henriques *et al.* 2010). Recent studies have also highlighted the functional interaction of RB regulatory pathways with insulin signaling (Hsieh *et al.* 2008; Mercader *et al.* 2009; Henriques *et al.* 2010). The direct targeting of signaling component gene promoters by RB family members may provide one means for a molecular linkage of these conserved pathways. To identify the true functional significance of these Rbf1-promoter interactions, more extensive analysis of *cis*-elements required for Rbf1 recruitment on individual promoters is required, as well as evaluation of the interplay of Rbf1 and these signaling pathways in a developmental context.

The integration of RB and insulin signaling would provide a means by which the sensitivity of this pathway would be controlled through differential expression of the insulin receptor, downstream kinases, and targets such as 4E-BP, a regulator of translation. Occupancy of target genes in the Wnt, Hh, EGFR, JNK, transforming growth factor-β, PI3K/Akt, insulin, AMPK, Notch, Hippo, JAK/STAT, nuclear factor-κB, and target of rapamycin pathways indicates that Rbf1 may have the potential to exert broad and concerted regulation of multiple signaling systems. Of the ~300 genes that we identified as core constituents of these pathways in *Drosophila*, approximately 46% exhibit significant promoter-proximal signals for Rbf1 occupancy, more than twice the frequency for genes at large. It is possible that Rbf1 controls all promoters involved in signaling pathways in a unified manner, or that some promoters are especially sensitive to the levels/activity of Rbf1 protein; determining how promoters of individual components of these pathways respond to this corepressor will be a first step to quantitatively modeling the interaction of these systems. One feature of the signal transduction genes bound by Rbf1 is the relative paucity of genes encoding extracellular signaling proteins; despite rich representation of receptors and intercellular components, very few ligands involved in the signaling pathways were among the observed targets of Rbf1. Perhaps Rbf1 is involved more in setting the cellular response curves of these systems than the levels of signals impinging on a cell.

Analysis of the physical targets of Rbf1 points to a richer suite of regulatory mechanisms for this protein’s output than has been previously indicated. Much attention has been focused on the role of reversible phosphorylation in regulation of RB activity, and other posttranslational forms of RB protein family regulation are well known, including proteolysis, methylation, and acetylation that control abundance, binding of regulatory factors, and nuclear localization. Our genomic analysis indicates that regulation of the RB pathway may extend to five levels of a functional hierarchy: through transcriptional control of cyclin and Hippo kinases that modify the protein and its function, through direct regulation of its own promoter, through regulation of proteins that work together with Rbf1 in some contexts to effect repression (MMB/dREAM and l(3)mbt), through regulation of downstream kinases that control meiotic and mitotic cell cycle, and finally through regulation of the levels of proteins that can functionally antagonize Rbf1 repression, namely the E(bx)/NURF301 chromatin remodeling factor. These types of regulatory linkages are unlikely to be restricted to *Drosophila*, thus the picture that emerges of RB pathways is one of tightly interwoven connections, where transcriptional links mediated through this family of proteins are likely to play important roles in adjusting the set points of numerous signaling pathways.

The almost exclusive genomic binding of Rbf1 very close to transcriptional start sites indicates that Rbf1 associates with genes in a very different sort of way compared to the binding of other transcriptional cofactors. Groucho and CtBP corepressor proteins are very broadly distributed, with no predisposition to localize to the transcriptional start sites (modENCODE). In contrast, most of the E2F2-containing MMB/dREAM components are tightly linked with basal promoters (Georlette *et al.* 2007). We hypothesize that E2F proteins may be short-range activators that only function when bound close to the basal promoter, similar to Sp1 activator proteins. To antagonize them, Rbf1 would be colocalized to these regions. Interestingly, in reanalyzing the data developed in Chicas *et al.* we note that approximately 70% of RB and p130 binding interactions are found within 1 kb of the transcriptional start sites of genes, suggesting that promoter proximity is a conserved feature of the RB family of corepressors (Chicas *et al.* 2010).

Our bioinformatic analysis of the Rbf1-bound regions clearly indicates that the regions occupied by Rbf1 are heterogeneous and that certain combinations of motifs are closely associated with functionally related genes (Figure 7). In some cases, these motifs may recruit proteins that bind adjacent to Rbf1 to provide specialized responses, similar to the way that modulatory proteins in mammals bind near E2F sites to functionally differentiate subclasses of these promoters (Jin *et al.* 2006; Freedman *et al.* 2009). The Rbf-associated MMB/dREAM complex, which contains several DNA-binding proteins, provides one example of this context: the complex binds to approximately 70% of the genes targeted by Rbf1 in the embryo (Table S7) (Georlette *et al.* 2007). Interestingly, few of these genes are involved in signaling pathways, suggesting that alternative Rbf1-containing complexes may form on these promoters. An additional feature of the signaling pathway genes is their lack of high-affinity E2F motifs, or sequences resembling the other three overrepresented motifs found on the rest of the Rbf1 targets, suggesting that E2F may interact with these promoters via non-canonical sites (Bieda *et al.* 2006; Xu *et al.* 2007). Our functional testing for E2F1 responsiveness (Figure S7) and Dyson lab’s recent publication (Korenjak *et al.* 2012) suggest that E2F2 and not E2F1 recruits Rbf1 to such promoters. A preliminary bioinformatic analysis of these signaling pathway promoter regions did not identify motifs common to the whole set; therefore, it is possible that there are subclasses of motifs that are involved in setting a transcriptional “grammar” for individual pathways.

In summary, our genomic identification of Rbf1 targets in the *Drosophila* embryo provides the first view of this important class of corepressor in a whole animal; we find that in addition to a core of conserved genes related to RB protein function in cell cycle and DNA replication, the Rbf1 occupied genes are distributed among a diversity of functions. The complexity of binding regions occupied by Rbf1 among different classes of genes strongly indicates that this corepressor is involved in gene regulation in very different contexts, interacting with promoters that are occupied by distinct types of transcription factors. Such complexity would allow the development of independently-controlled groups of Rbf1 target genes. Much work remains in deciphering the “promoter grammar” of these regulatory regions. Most intriguingly, a high degree of enrichment of genes for conserved signaling pathways suggests that Rbf1 is directly involved in setting levels of components of these systems at multiple points; such regulation would change the sensitivity of signaling, which may vary from tissue to tissue. Identifying the functional significance of RB interactions with genes from these pathways will clarify new pathways of regulation of importance in development and disease.

## Supplementary Material

Supporting Information
