# Supplementary material for: Evidence for Autoregulation and Cell Signaling Pathway Regulation From Genome-Wide Binding of the Drosophila Retinoblastoma Protein
Source: G3 (Bethesda). 2012 Nov 1;2(11):1459–72. doi: 10.1534/g3.112.004424 (PMC3484676; doi:10.1534/g3.112.004424)
Supplement: Supporting Information [file supp_2_11_1459__index.html]

Supporting Information 

# Evidence for Autoregulation and Cell Signaling Pathway Regulation From Genome-Wide Binding of the *Drosophila* Retinoblastoma Protein

## Supporting Information for Acharya *et al.*, 2012

**Files in this Data Supplement:**

- Supporting Information - Figures S1-S7, File S1, and Tables S1-S8 (PDF, 1.1 MB)
- Figure S1 - Enrichment of Rbf1-bound promoters peaks at 12-18 hr (PDF, 154 KB)
- Figure S2 - Validation of selected promoters for Rbf1 occupancy (PDF, 446 KB)
- Figure S3 - Validation of specificity of Rbf1 antibodies (PDF, 203 KB)
- Figure S4 - Repression of *InR* and *Rab23* promoters by Rbf1 (PDF, 112 KB)
- Figure S5 - Determination of threshold for motif analysis (PDF, 235 KB)
- Figure S6 - Prevalence of E2F-, DREF- and RAM-like motifs on Rbf-1 bound and not bound promoter regions (PDF, 156 KB)
- Figure S7 - E2F responsiveness of promoters of selected genes in signaling pathways (PDF, 122 KB)
- File S1 - Supporting Materials and Methods (PDF, 68 KB)
- Table S1 - Peaks bound by Rbf1 protein (.xls, 977 KB)
- Table S2 - Genes in diverse signaling pathways targeted by Rbf1 (.xls, 45 KB)
- Table S3 - GO categories of genes described in Figure 5 (.xls, 153 KB)
- Table S4 - Enrichment of E2F, DREF and RAM motifs in Rbf1-bound promoters (.xls, 18 KB)
- Table S5 - Enrichment of motifs in specific subclasses of Rbf1 target genes (.xls, 20 KB)
- Table S6 - List of oligonucleotides used for PCR (.xls, 29 KB)
- Table S7 - List of common physical targets of Rbf1 and dREAM complex (.xls, 176 KB)
- Table S8 - List of signaling pathways genes analyzed in this study (.xls, 219 KB)
