# Supplementary material for: Evidence for Autoregulation and Cell Signaling Pathway Regulation From Genome-Wide Binding of the Drosophila Retinoblastoma Protein
Source: G3 (Bethesda). 2012 Nov 1;2(11):1459–72. doi: 10.1534/g3.112.004424 (PMC3484676; doi:10.1534/g3.112.004424)
Supplement: Supporting Information [file supp_2.11.1459_004424SI.pdf]

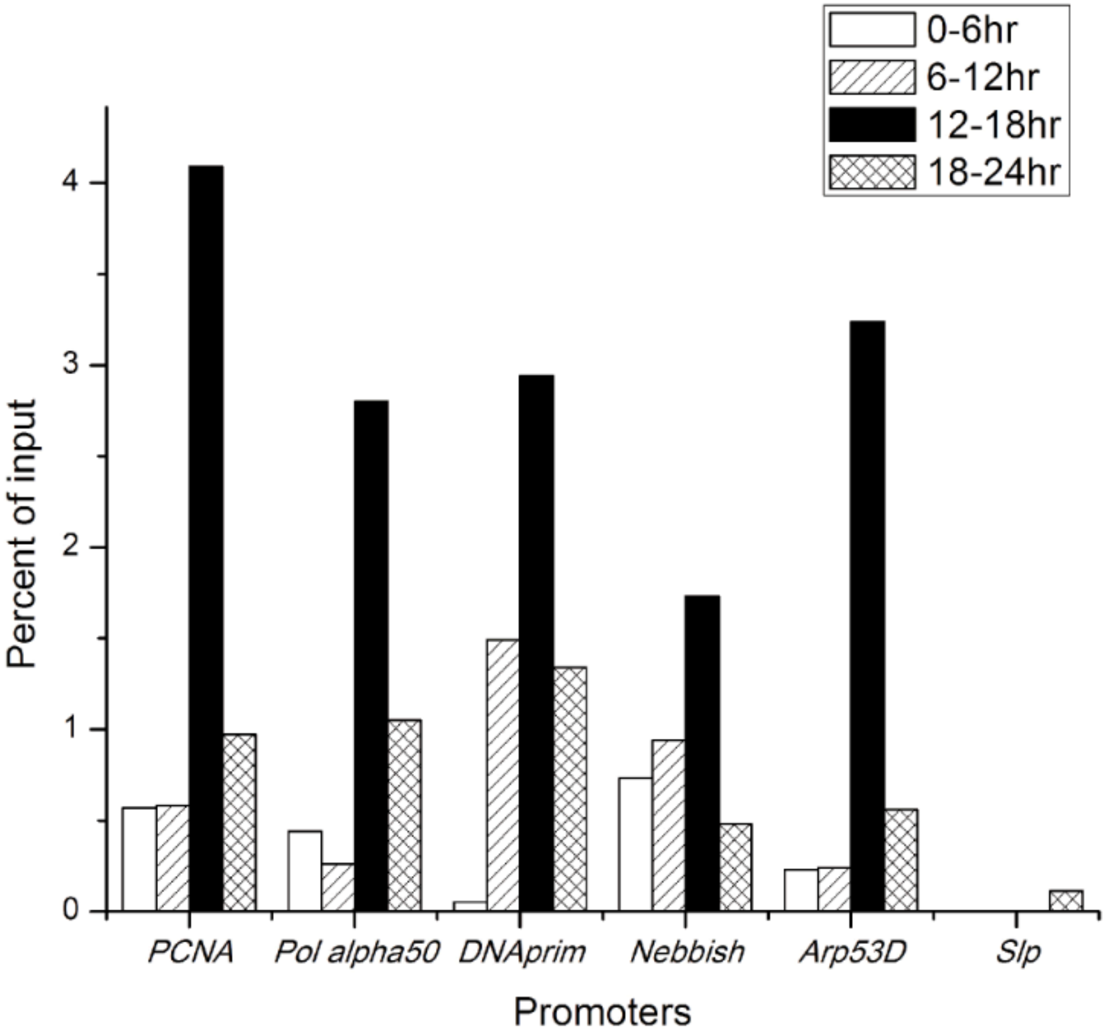

Figure S1 Enrichment of Rbf1-bound promoters peaks at 12-18 hr. Quantitation of enrichment of Rbf1-bound promoters shows that the dynamic promoter occupancy of Rbf1 is maximum at 12-18 hr. The PCR products shown in Figure 1 were measured on a Fuji LAS3000 imager and quantitated using Multi Gauge software (Fuji).

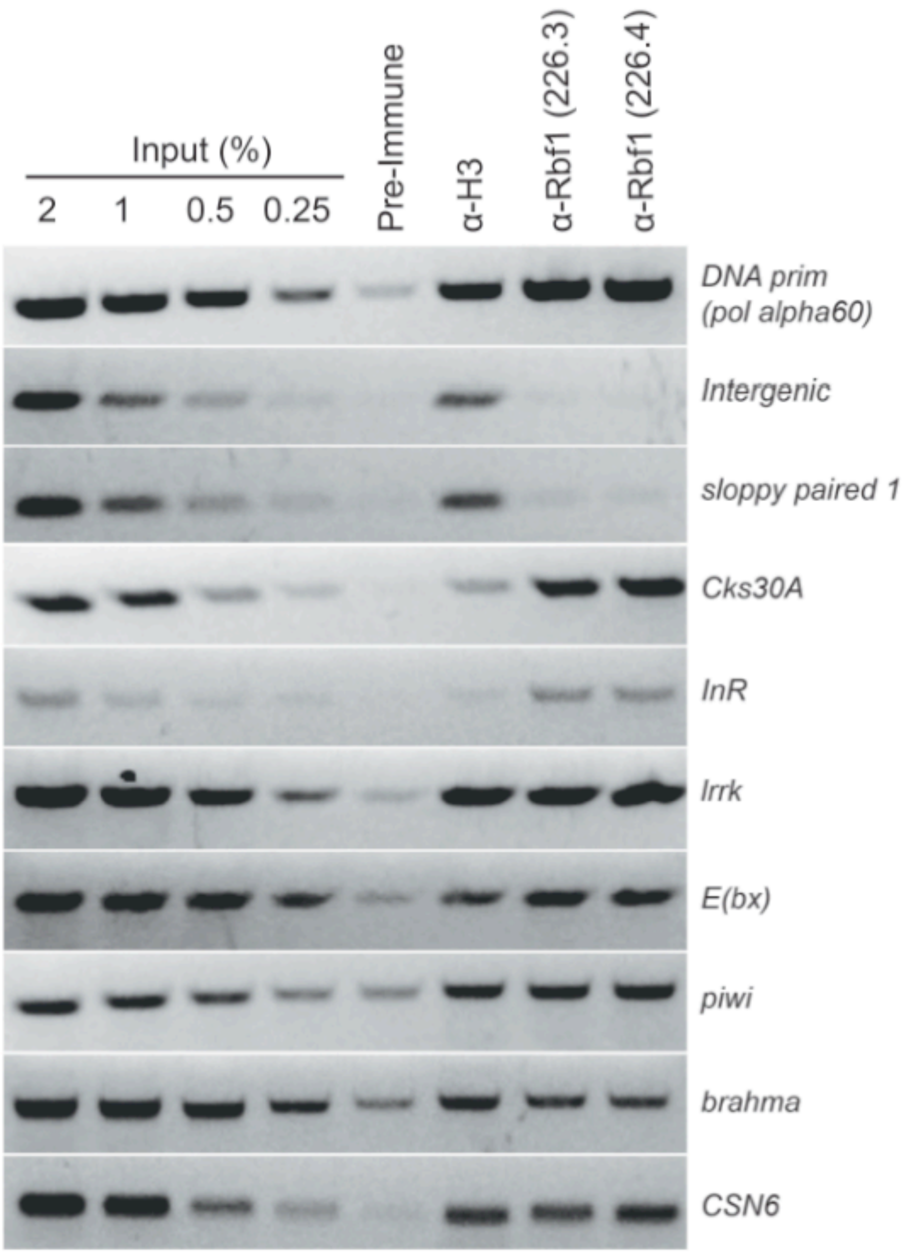

Figure S2 Validation of selected promoters for Rbf1 occupancy. To independently assess enrichment of Rbf1 on novel target genes, several genes were selected and their enrichment in ChIPed chromatin was tested by PCR. *DNA prim* is a positive control; the intergenic region on chromosome 3 and *sloppy paired 1* are negative controls. The enrichment of the Rbf1 target gene promoters tested is significantly above the background. "Preimmune", serum from the rabbit used for later generation of α-Rbf1 antibody; "α-H3", anti-histone H3 antibody; "α-Rbf1 226.3" and "α-Rbf1 226.4", different bleeds of rabbit anti-Rbf1 antibodies.

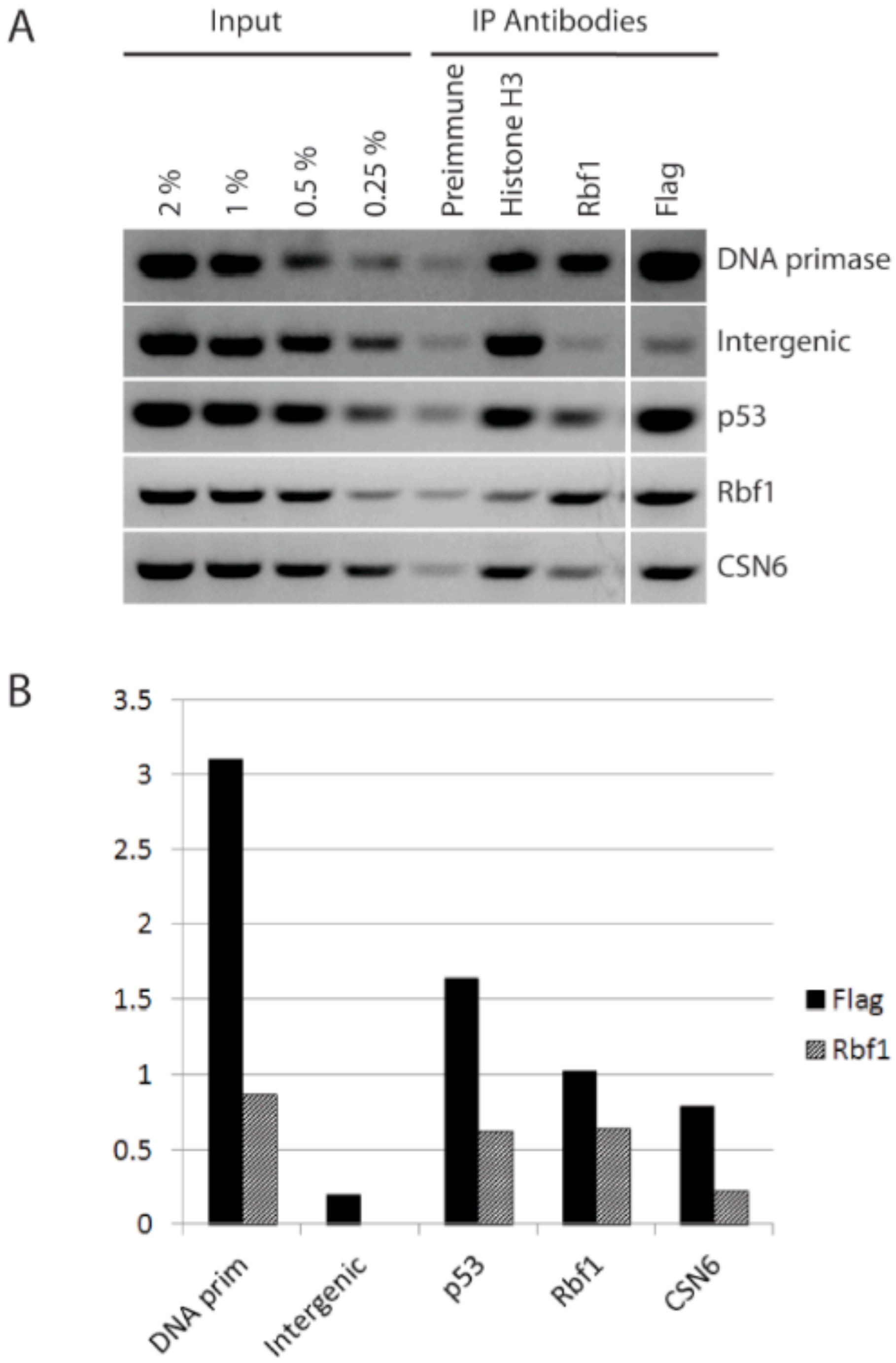

Figure S3 Validation of specificity of Rbf1 antibodies. (A) To assess the specificity of Rbf1 antibodies, a ChIP experiment was performed with embryos from transgenic flies harboring Flag epitope tagged Rbf1. Several genes identified in the ChIP-seq analysis along with a previously known target (*DNA primase)* and intergenic region were selected for PCR. A similar significant enrichment of the Rbf1 target gene promoters was noted for each antibody. "Preimmune", serum from the rabbit used for later generation of α-Rbf1 antibody; "α-H3", anti-histone H3 antibody; "α-Rbf1", rabbit anti-Rbf1 antibody; "Flag", anti-Flag antibody. (B) Quantitation of the signals in (A).

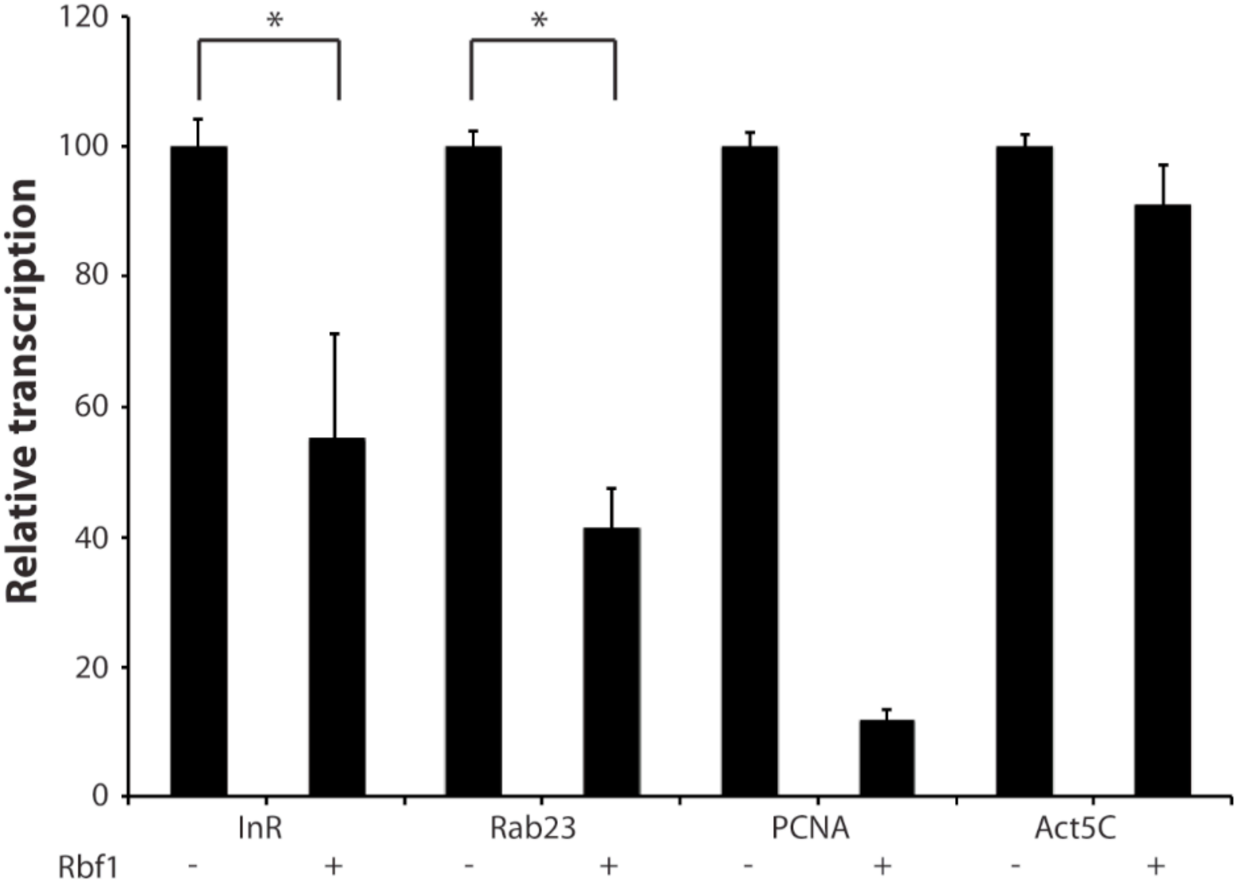

Figure S4 Repression of *InR* and *Rab23* promoters by Rbf1. *Drosophila* S2 cells were cotransfected with *InR, Rab23*, *PCNA,* or *Act5C* luciferase reporters, with (+) or without (-) an Rbf1-expressing plasmid. Cells were harvested 72 h after transfection, and luciferase assay was performed. Results of four (*Act5c* luciferase), and six (*InR, Rab23,* and *PCNA* luciferase) biological replicates with three technical replicates each were pooled. Asterisks indicate $p < 0.0001$.

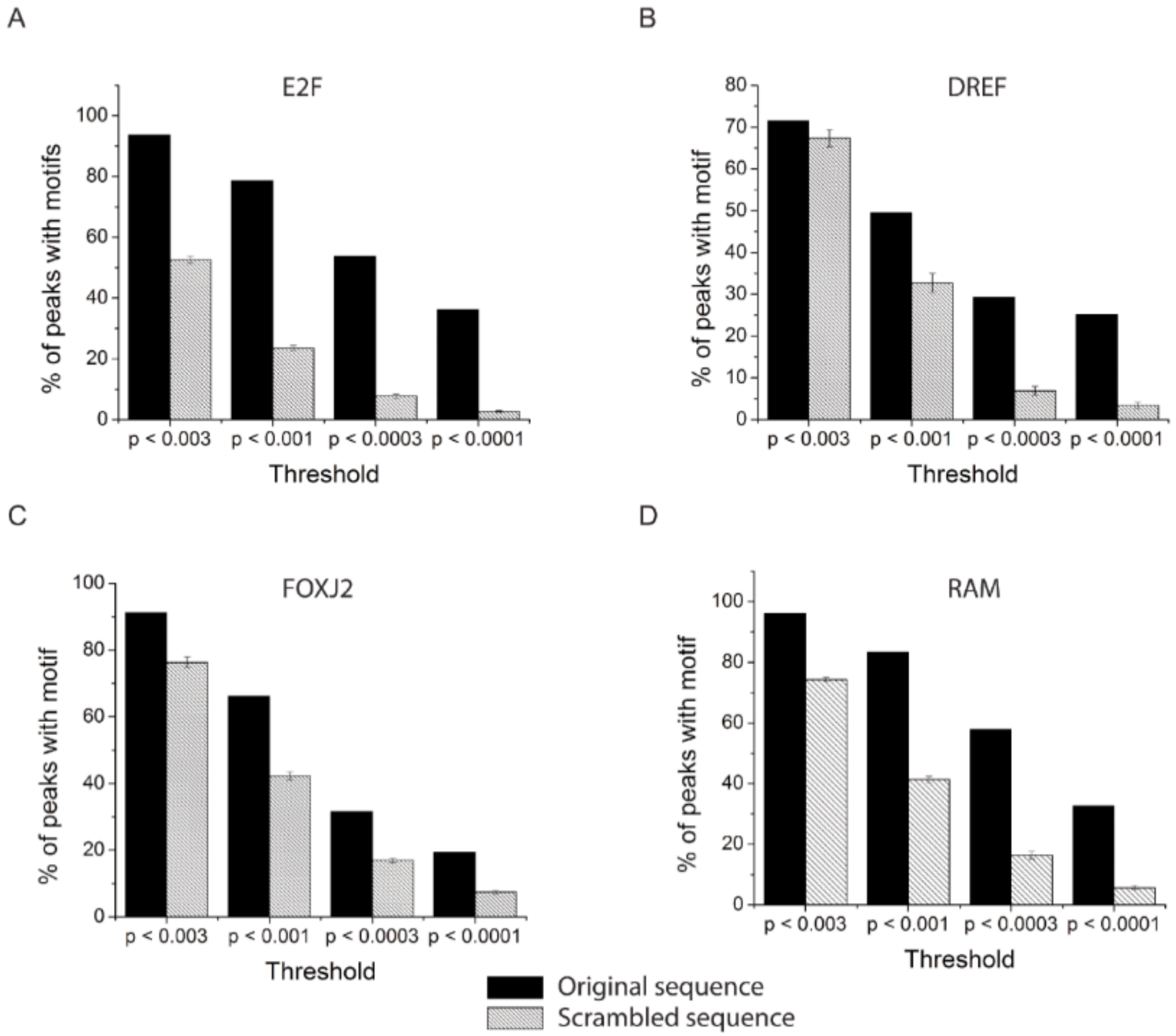

Figure S5 Determination of threshold for motif analysis. To determine optimal p values to minimize false positive calls and provide reasonable sensitivity, MAST analysis was performed on sequences under peaks with different thresholds for E2F, DREF, FOXJ2 and RAM motifs (A-D). The analysis was repeated five times on sequences of identical A/T composition that had been scrambled. The threshold $p < 0.0001$ showed the highest fold enrichment, thus further analyses (in Figure 6 B, C and D) were carried out with this value.

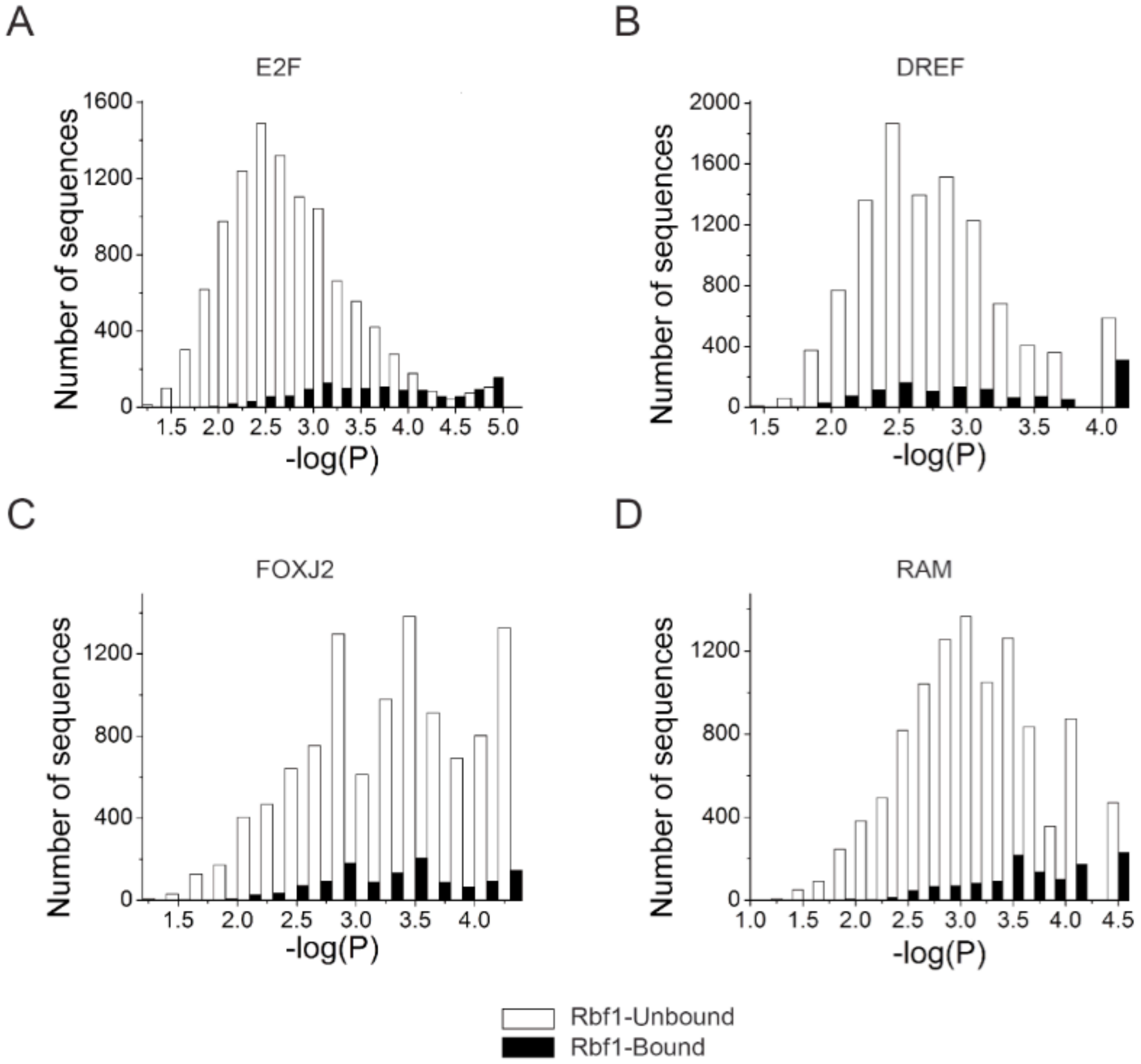

Figure S6 Prevalence of E2F-, DREF- and RAM-like motifs on Rbf-1 bound and not bound promoter regions. Scores for each Rbf1-bound peak (black bars) and Rbf1-unbound 200 bp promoter region centered at -200 bp (white bars) obtained from MAST analysis show that Rbf1-bound promoters have better E2F, DREF and RAM motifs (in each case, Wilcoxon rank sum test $p < 2.2e-16$). However, there was no significant difference in scores for FOXJ2 motifs in Rbf1-bound or unbound promoters (Wilcoxon rank sum test $p = 0.16$). The significant enrichment of weak E2F, DREF, and RAM sites among bound genes suggests that there may be a higher fraction of Rbf1-bound promoters that utilize these proteins than indicated by the use of our stringent cutoff criteria. An alternative statistical analysis of the peaks indicated that the prevalence of these motifs in Rbf1-bound and -unbound promoters is significant (Supplementary Table IV).

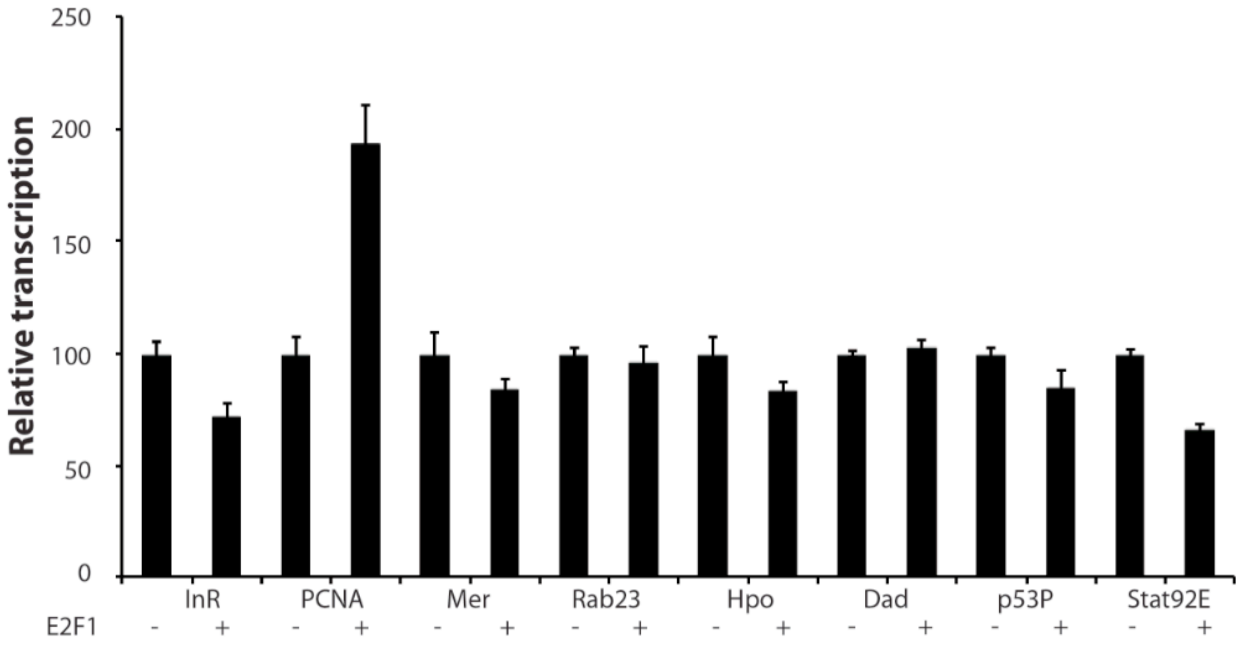

Figure S7 E2F responsiveness of promoters of selected genes in signaling pathways. *Drosophila* S2 cells were cotransfected with *InR, PCNA, Merlin, Rab23, Hippo, Dad, p53-proximal,* or *Stat92E* luciferase reporters, with (+) or without (-) a plasmid overexpressing E2F1. Only *PCNA* luciferase expression was elevated by E2F1.

File S1

Supporting Materials and Methods

Chromatin Immunoprecipitation

For preparation of chromatin, embryos were collected, dechorionated with bleach and placed in a 50-ml tube with 9.4 ml of phosphate-buffered saline (137 mM NaCl, 2.7 mM KCl, 10 mM Na_2HPO_4, 1.76 mM KH_2PO_4, pH 7.4). 0.6 ml of freshly prepared 25mM DSP [dithiobis(succinimidyl propionate)] was added to the embryos in this buffer, and the tube was shaken vigorously for 30 min at room temperature, centrifuged at 1500 rpm in a Beckman Allegra 6R clinical centrifuge for 5 min and supernatant was removed. Embryos were fixed for 15 min with vigorous shaking in a 50 ml tube in 9.2 ml crosslinking buffer (50mM HEPES [pH 7.6], 1mM EDTA, 0.5mM EGTA, 100mM NaCl), 0.81 ml of 37% formaldehyde and 30 ml heptane. The cross-linking reaction was stopped with 25 ml stop buffer (0.125M glycine, 0.01% Triton X-100 in phosphate-buffered saline [PBS]) while the tube was shaken vigorously for 15 min at room temperature. The supernatant was removed and the embryos were washed in 10 ml embryo wash buffer (10 mM HEPES[pH 7.6], 200 mM NaCl, 1 mM EDTA, 0.5 mM EGTA, 0.01% Triton X-100) for 10 min with vigorous agitation at room temperature. The supernatant was removed and embryos were resuspended in 5 ml of sonication buffer (10 mM HEPES[pH 7.6], 1mM EDTA, 0.5 mM EGTA, 0.1% sodium deoxycholate), transferred to a 15-ml tube and a proteinase inhibitor tablet (Roche complete mini, 11-836-153-001) was added. The embryos were sonicated for 20 s (60% duty cycle) and cooled on ice for 30 s a total of 12 times followed by three 30 s sonication and 30 s cooling cycles, using a Branson sonicator. Crude chromatin was centrifuged at 14000 rpm in a microcentrifuge at 4°C and supernatant was transferred to a 15-ml tube. An equal volume of room temperature 2X radioimmunoprecipitation assay (RIPA) buffer (2% Triton X-100, 280 mM NaCl, 20 mM Tris-HCl [pH 8.0], 2 mM EDTA) was added. The chromatin was precleared by adding 10 µl/ml of a 50% slurry containing an equal mixture of agarose beads coupled to protein A and protein G (Millipore; equal volume of protein A and G beads were mixed) previously washed three times with 1 X RIPA buffer and blocked 2 hr at room temperature with 0.1 mg/ml bovine serum albumin and 0.2 mg/ml salmon sperm DNA. For immunoprecipitations, 1 ml of precleared chromatin was incubated with 5 µl preimmune, 5 µl Rbf1 antibody [24], or 2 µl H3 antibody (Abcam, Cambridge, MA; 0.4 µg/ µl) overnight at 4°C. After overnight incubation, the samples were centrifuged at 14000 rpm in a microcentrifuge for 10 minutes at 4°C. Supernatant was taken in microcentrifuge tubes and 40 µl blocked beads (as described above) were added to each sample and incubated for 2 h (with rotating) at 4°C. The beads were centrifuged in a microcentrifuge at 1000 rpm for 1 min, washed twice with 1ml ice cold low-salt buffer (0.1% SDS, 1% Triton X-100, 2 mM EDTA, 20 mM Tris-Cl [pH 8.0], 150 mM NaCl), twice with ice cold high-salt buffer (0.1% SDS, 1% Triton X-100, 2 mM EDTA, 20 mM Tris-Cl [pH 8.0], 500 mM NaCl) and twice with Tris-EDTA (10 mM Tris [pH 8.0], 1 mM EDTA). Chromatin was eluted at room temperature with 250 µl elution buffer (1% SDS, 0.1 M $NaHCO_3$) for 15 min without shaking. Beads were centrifuged, the supernatant was transferred to a microcentrifuge tube, a second elution was performed, and supernatants were combined. 25 µl of 4M NaCl was added, and cross-links were reversed overnight at 65°C. The eluates were then incubated with 1 µl RNase A (10 mg/ml) at 37°C for 30 min. 10 µl 0.5 M EDTA, 20 µl 1 M Tris-Cl (pH 6.5), and 1 µl proteinase K (20 mg/ml) were then added and tubes incubated at 42°C for 1 h. DNA was extracted with phenol-chloroform and precipitated with equal volume of isopropanol, 3 M sodium acetate (final concentration 0.3M) and GlycoBlue pellet paint by centrifuging at 14000 rpm at 4°C. The pellets were carefully washed once with 70% ethanol, air dried and resuspended in 40 µl water. 2 µl of each ChIP sample was used for 31 cycles of PCR for 0-6 hr embryos and 28 cycles of PCR for the DNA from other time points. The oligonucleotides used for PCR are listed in Table S8.

Supporting Tables

Tables S1-S8 are available for download at http://www.g3journal.org/lookup/suppl/doi:10.1534/g3.112.004424/-/DC1.

Table S1 Peaks bound by Rbf1 protein. Class A indicates peaks identified in both biological replicates (values are shown from experiment 1); Class B indicates peaks identified only in experiment 1; and Class C indicates the few peaks identified only in experiment 2.

Table S2 Genes in diverse signaling pathways targeted by Rbf1. About 40% of all conserved signaling pathway genes in Drosophila are targeted by Rbf1. Genes are listed in numerical order by CG number.

Table S3 GO categories of genes described in Figure 5. Genes in related GO categories were manually combined into super-GO categories. Genes in each category are listed in numerical order by CG number.

Table S4 Enrichment of E2F, DREF and RAM motifs in Rbf1-bound promoters. MAST analysis with $p < 0.0001$ was performed on Rbf1-bound and Rbf1-unbound promoters. The table shows number of promoters with motifs. The presence of FOXJ2 motifs in Rbf1-bound promoters is similar to Rbf1-unbound promoters. P-values for Fisher's exact test are shown in the last column.

Table S5 Enrichment of motifs in specific subclasses of Rbf1 target genes. Proportion of genes in each category with different motifs as illustrated in Figure 7.

Table S6 List of oligonucleotides used for PCR.

Table S7 List of common physical targets of Rbf1 and dREAM complex. Genes bound by dREAM complex proteins are derived in Georlette et al, 2007.

Table S8 List of signaling pathways genes analyzed in this study. The first subtable indicates occupancy of all 295 signaling pathway genes by Rbf1, RB, and p130. The subsequent subtables show whether Rbf1 binds to RB/p130 targets, divided by individual pathway.
