# Supplementary material for: Evidence for Autoregulation and Cell Signaling Pathway Regulation From Genome-Wide Binding of the Drosophila Retinoblastoma Protein
Source: G3 (Bethesda). 2012 Nov 1;2(11):1459–72. doi: 10.1534/g3.112.004424 (PMC3484676; doi:10.1534/g3.112.004424)
Supplement: Supporting Information [file supp_2.11.1459_FigureS1.pdf]

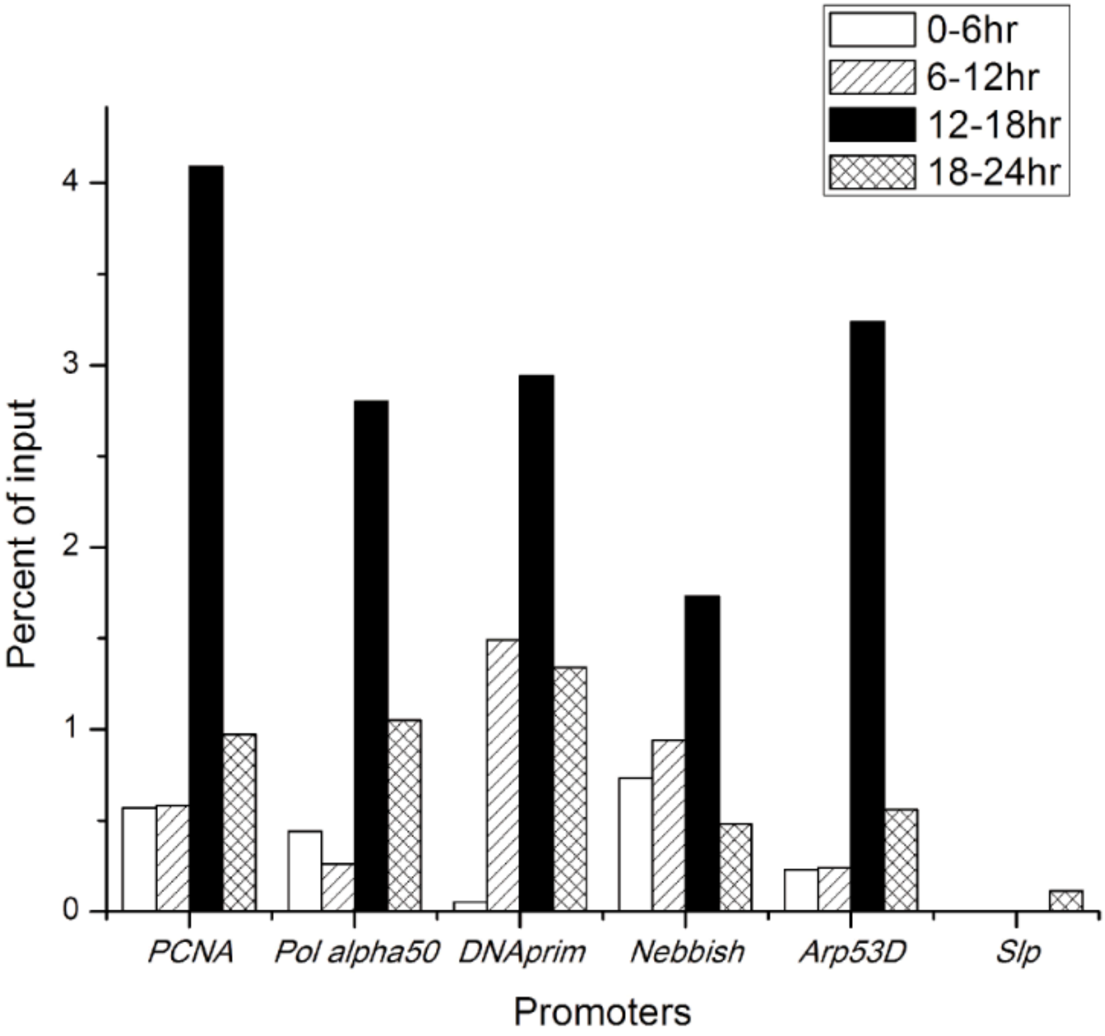

Figure S1 Enrichment of Rbf1-bound promoters peaks at 12-18 hr. Quantitation of enrichment of Rbf1-bound promoters shows that the dynamic promoter occupancy of Rbf1 is maximum at 12-18 hr. The PCR products shown in Figure 1 were measured on a Fuji LAS3000 imager and quantitated using Multi Gauge software (Fuji).
