# Supplementary material for: Evidence for Autoregulation and Cell Signaling Pathway Regulation From Genome-Wide Binding of the Drosophila Retinoblastoma Protein
Source: G3 (Bethesda). 2012 Nov 1;2(11):1459–72. doi: 10.1534/g3.112.004424 (PMC3484676; doi:10.1534/g3.112.004424)
Supplement: Supporting Information [file supp_2.11.1459_FigureS2.pdf]

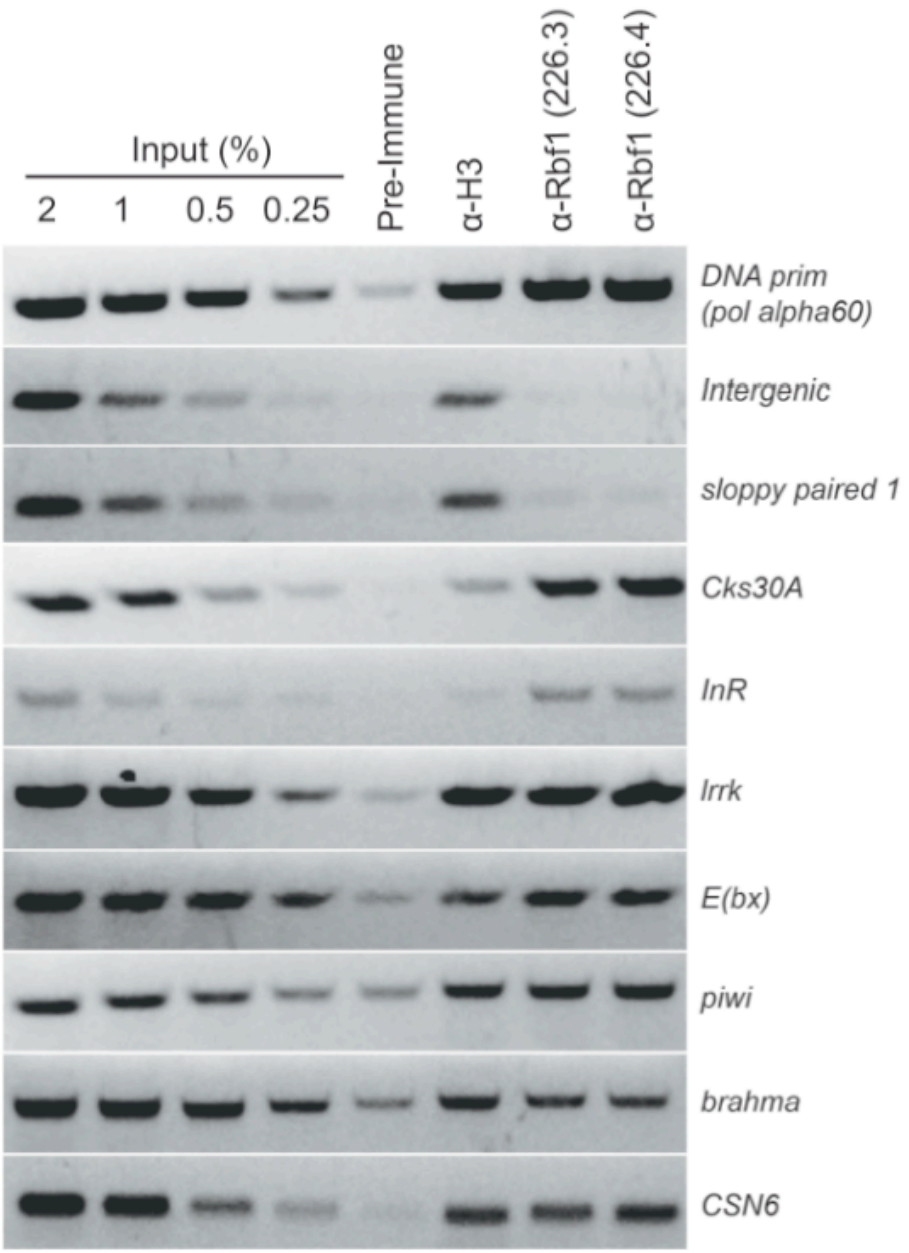

Figure S2 Validation of selected promoters for Rbf1 occupancy. To independently assess enrichment of Rbf1 on novel target genes, several genes were selected and their enrichment in ChIPed chromatin was tested by PCR. *DNA prim* is a positive control; the intergenic region on chromosome 3 and *sloppy paired 1* are negative controls. The enrichment of the Rbf1 target gene promoters tested is significantly above the background. “Preimmune”, serum from the rabbit used for later generation of α-Rbf1 antibody; “α-H3”, anti-histone H3 antibody; “α-Rbf1 226.3” and “α-Rbf1 226.4”, different bleeds of rabbit anti-Rbf1 antibodies.
