# Supplementary material for: Evidence for Autoregulation and Cell Signaling Pathway Regulation From Genome-Wide Binding of the Drosophila Retinoblastoma Protein
Source: G3 (Bethesda). 2012 Nov 1;2(11):1459–72. doi: 10.1534/g3.112.004424 (PMC3484676; doi:10.1534/g3.112.004424)
Supplement: Supporting Information [file supp_2.11.1459_FigureS3.pdf]

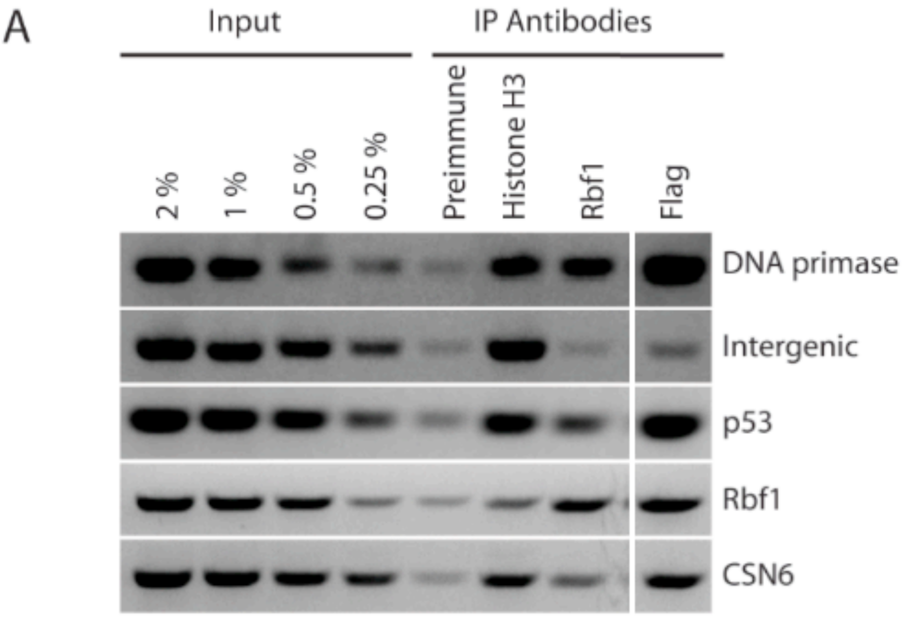

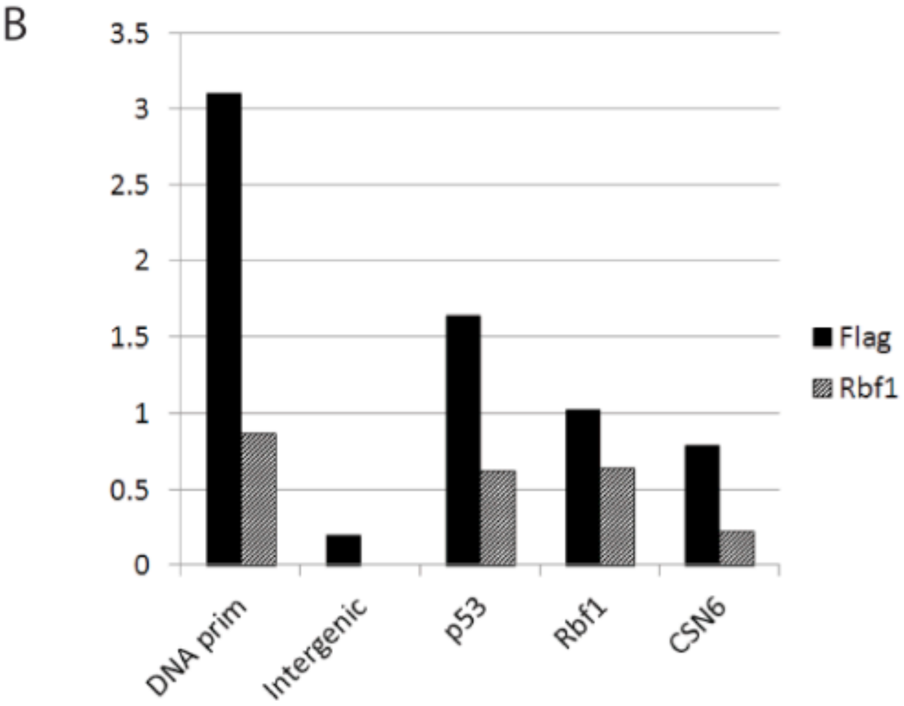

Figure S3 Validation of specificity of Rbf1 antibodies. (A) To assess the specificity of Rbf1 antibodies, a ChIP experiment was performed with embryos from transgenic flies harboring Flag epitope tagged Rbf1. Several genes identified in the ChIP-seq analysis along with a previously known target (*DNA primase)* and intergenic region were selected for PCR. A similar significant enrichment of the Rbf1 target gene promoters was noted for each antibody. "Preimmune", serum from the rabbit used for later generation of α-Rbf1 antibody; "α-H3", anti-histone H3 antibody; "α-Rbf1", rabbit anti-Rbf1 antibody; "Flag", anti-Flag antibody. (B) Quantitation of the signals in (A).
