# Supplementary material for: Evidence for Autoregulation and Cell Signaling Pathway Regulation From Genome-Wide Binding of the Drosophila Retinoblastoma Protein
Source: G3 (Bethesda). 2012 Nov 1;2(11):1459–72. doi: 10.1534/g3.112.004424 (PMC3484676; doi:10.1534/g3.112.004424)
Supplement: Supporting Information [file supp_2.11.1459_FigureS4.pdf]

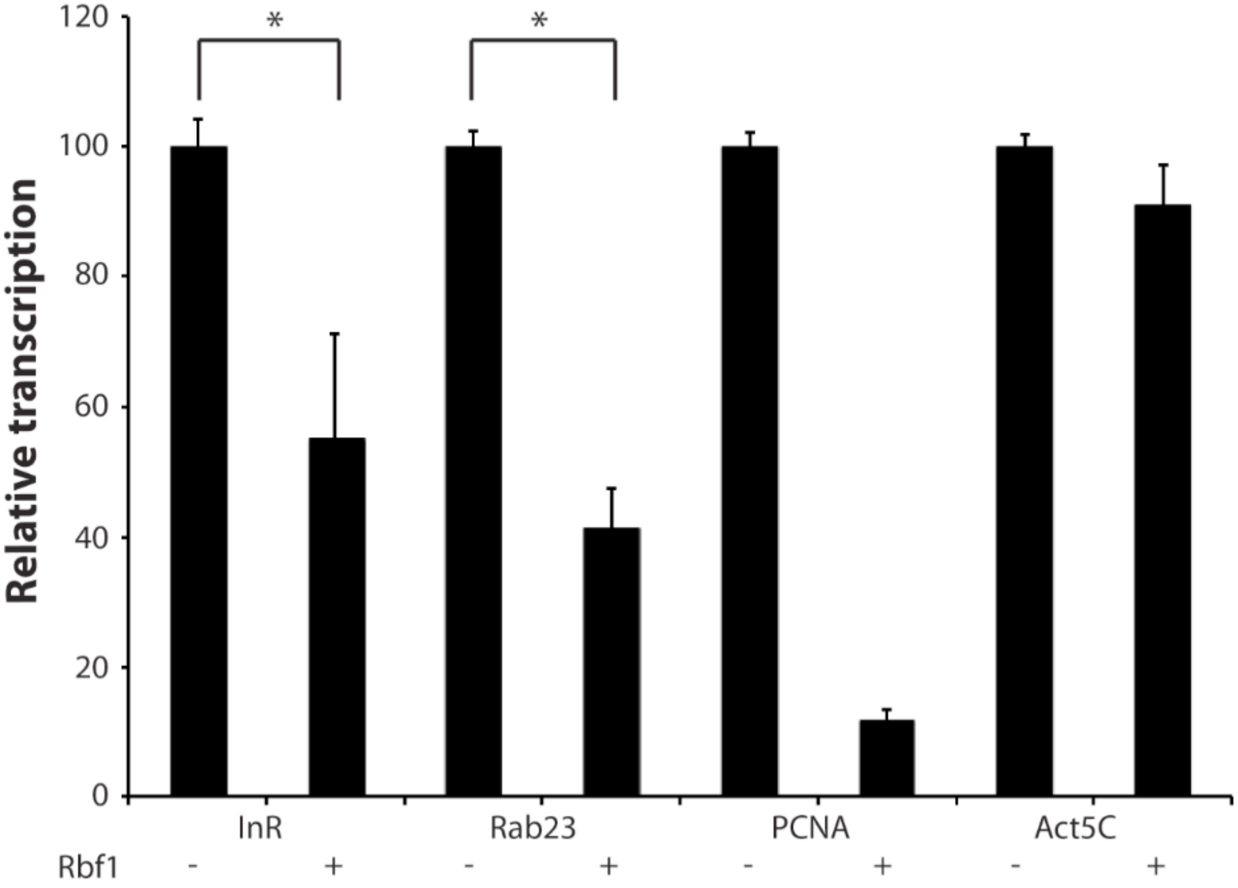

Figure S4 Repression of *InR* and *Rab23* promoters by Rbf1. *Drosophila* S2 cells were cotransfected with *InR, Rab23*, *PCNA,* or *Act5C* luciferase reporters, with (+) or without (-) an Rbf1-expressing plasmid. Cells were harvested 72 h after transfection, and luciferase assay was performed. Results of four (*Act5c* luciferase), and six (*InR, Rab23,* and *PCNA* luciferase) biological replicates with three technical replicates each were pooled. Asterisks indicate $p < 0.0001$.
