# Supplementary material for: Evidence for Autoregulation and Cell Signaling Pathway Regulation From Genome-Wide Binding of the Drosophila Retinoblastoma Protein
Source: G3 (Bethesda). 2012 Nov 1;2(11):1459–72. doi: 10.1534/g3.112.004424 (PMC3484676; doi:10.1534/g3.112.004424)
Supplement: Supporting Information [file supp_2.11.1459_FigureS5.pdf]

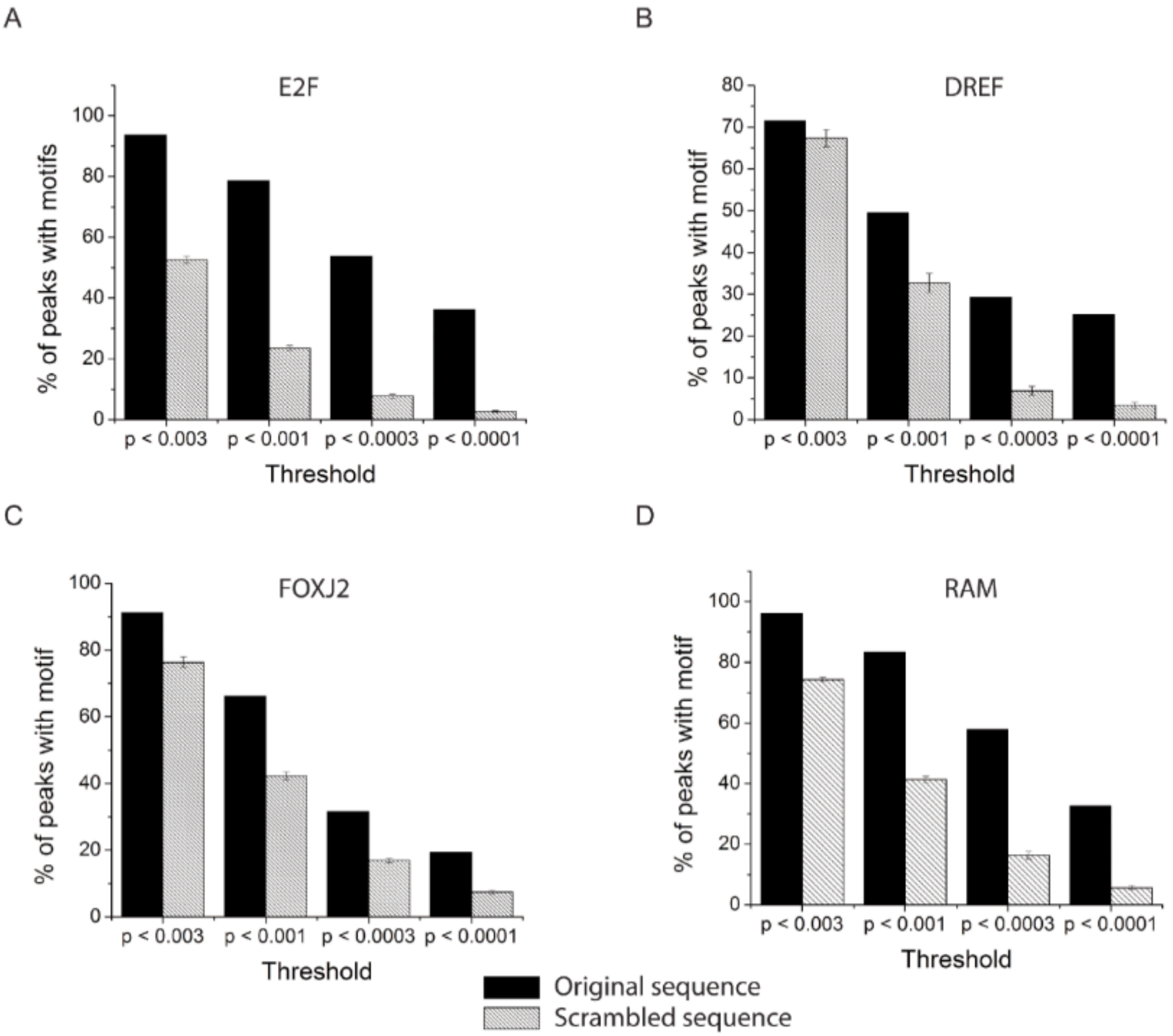

Figure S5 Determination of threshold for motif analysis. To determine optimal p values to minimize false positive calls and provide reasonable sensitivity, MAST analysis was performed on sequences under peaks with different thresholds for E2F, DREF, FOXJ2 and RAM motifs (A-D). The analysis was repeated five times on sequences of identical A/T composition that had been scrambled. The threshold $p < 0.0001$ showed the highest fold enrichment, thus further analyses (in Figure 6 B, C and D) were carried out with this value.
