# Supplementary material for: Evidence for Autoregulation and Cell Signaling Pathway Regulation From Genome-Wide Binding of the Drosophila Retinoblastoma Protein
Source: G3 (Bethesda). 2012 Nov 1;2(11):1459–72. doi: 10.1534/g3.112.004424 (PMC3484676; doi:10.1534/g3.112.004424)
Supplement: Supporting Information [file supp_2.11.1459_FigureS6.pdf]

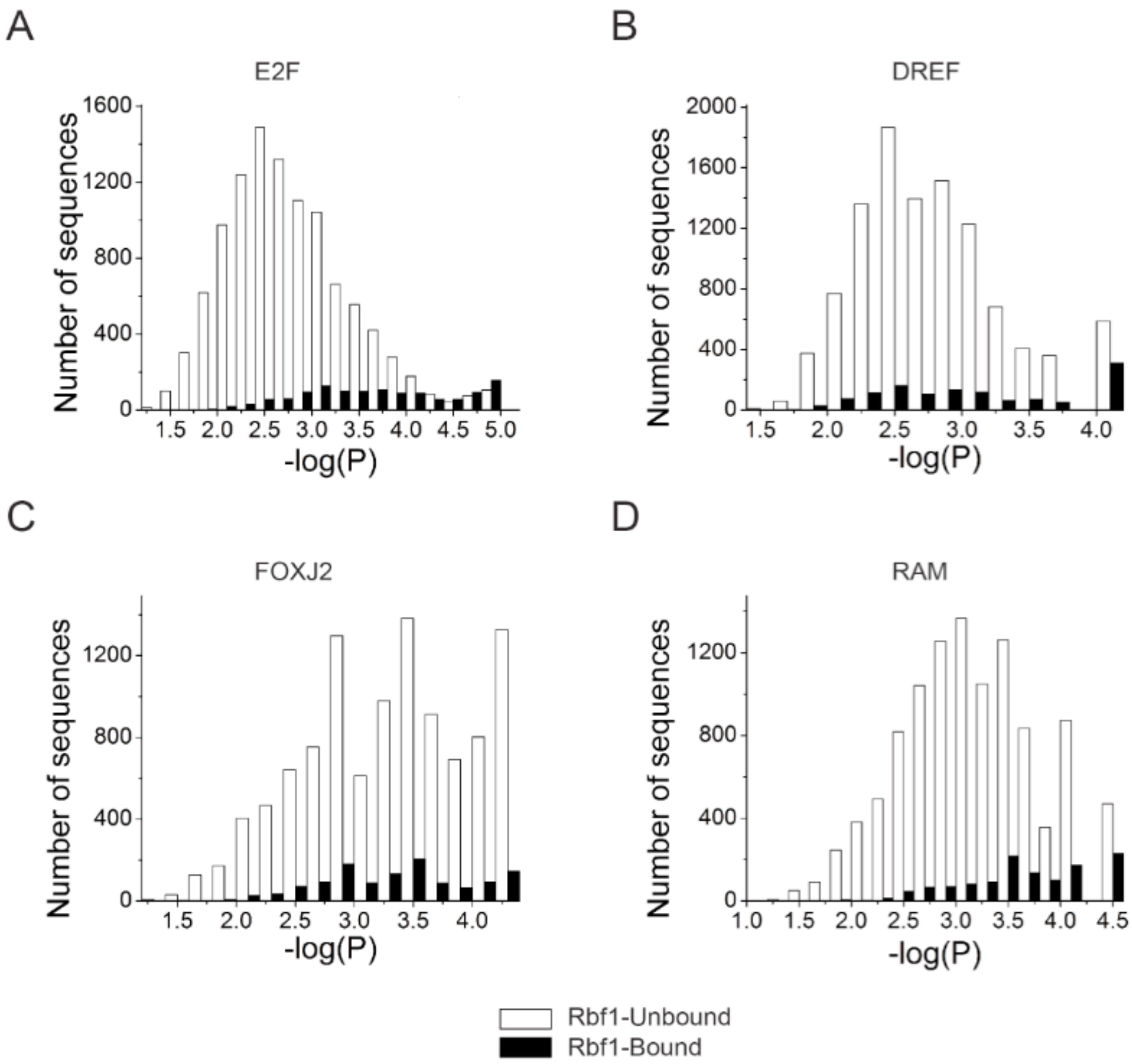

Figure S6 Prevalence of E2F-, DREF- and RAM-like motifs on Rbf-1 bound and not bound promoter regions. Scores for each Rbf1-bound peak (black bars) and Rbf1-unbound 200 bp promoter region centered at -200 bp (white bars) obtained from MAST analysis show that Rbf1-bound promoters have better E2F, DREF and RAM motifs (in each case, Wilcoxon rank sum test $p < 2.2e-16$). However, there was no significant difference in scores for FOXJ2 motifs in Rbf1-bound or unbound promoters (Wilcoxon rank sum test $p = 0.16$). The significant enrichment of weak E2F, DREF, and RAM sites among bound genes suggests that there may be a higher fraction of Rbf1-bound promoters that utilize these proteins than indicated by the use of our stringent cutoff criteria. An alternative statistical analysis of the peaks indicated that the prevalence of these motifs in Rbf1-bound and -unbound promoters is significant (Supplementary Table IV).
