# Supplementary material for: Evidence for Autoregulation and Cell Signaling Pathway Regulation From Genome-Wide Binding of the Drosophila Retinoblastoma Protein
Source: G3 (Bethesda). 2012 Nov 1;2(11):1459–72. doi: 10.1534/g3.112.004424 (PMC3484676; doi:10.1534/g3.112.004424)
Supplement: Supporting Information [file supp_2.11.1459_FigureS7.pdf]

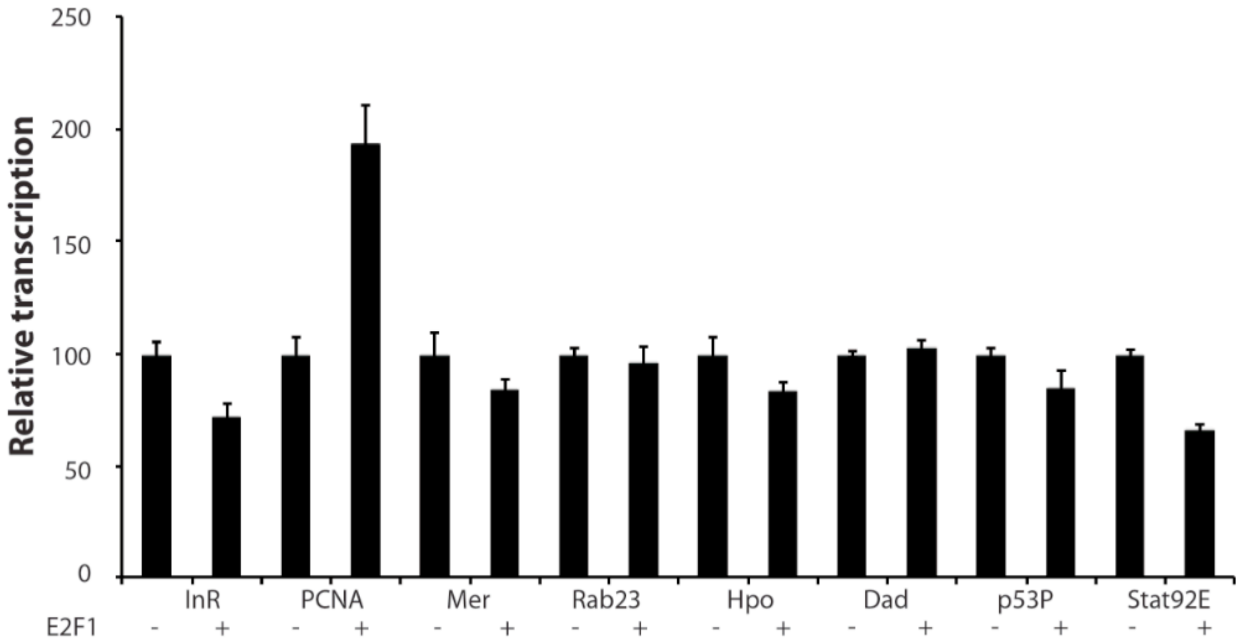

Figure S7 E2F responsiveness of promoters of selected genes in signaling pathways. *Drosophila* S2 cells were cotransfected with *InR, PCNA, Merlin, Rab23, Hippo, Dad, p53-proximal,* or *Stat92E* luciferase reporters, with (+) or without (-) a plasmid overexpressing E2F1. Only *PCNA* luciferase expression was elevated by E2F1.
