# Supplementary material for: Evidence for Autoregulation and Cell Signaling Pathway Regulation From Genome-Wide Binding of the Drosophila Retinoblastoma Protein
Source: G3 (Bethesda). 2012 Nov 1;2(11):1459–72. doi: 10.1534/g3.112.004424 (PMC3484676; doi:10.1534/g3.112.004424)
Supplement: Supporting Information [file supp_2.11.1459_FileS1.pdf]

File S1

Supporting Materials and Methods

Chromatin Immunoprecipitation

For preparation of chromatin, embryos were collected, dechorionated with bleach and placed in a 50-ml tube with 9.4 ml of phosphate-buffered saline (137 mM NaCl, 2.7 mM KCl, 10 mM Na_2HPO_4, 1.76 mM KH_2PO_4, pH 7.4). 0.6 ml of freshly prepared 25mM DSP [dithiobis(succinimidyl propionate)] was added to the embryos in this buffer, and the tube was shaken vigorously for 30 min at room temperature, centrifuged at 1500 rpm in a Beckman Allegra 6R clinical centrifuge for 5 min and supernatant was removed. Embryos were fixed for 15 min with vigorous shaking in a 50 ml tube in 9.2 ml crosslinking buffer (50mM HEPES [pH 7.6], 1mM EDTA, 0.5mM EGTA, 100mM NaCl), 0.81 ml of 37% formaldehyde and 30 ml heptane. The cross-linking reaction was stopped with 25 ml stop buffer (0.125M glycine, 0.01% Triton X-100 in phosphate-buffered saline [PBS]) while the tube was shaken vigorously for 15 min at room temperature. The supernatant was removed and the embryos were washed in 10 ml embryo wash buffer (10 mM HEPES[pH 7.6], 200 mM NaCl, 1 mM EDTA, 0.5 mM EGTA, 0.01% Triton X-100) for 10 min with vigorous agitation at room temperature. The supernatant was removed and embryos were resuspended in 5 ml of sonication buffer (10 mM HEPES[pH 7.6], 1mM EDTA, 0.5 mM EGTA, 0.1% sodium deoxycholate), transferred to a 15-ml tube and a proteinase inhibitor tablet (Roche complete mini, 11-836-153-001) was added. The embryos were sonicated for 20 s (60% duty cycle) and cooled on ice for 30 s a total of 12 times followed by three 30 s sonication and 30 s cooling cycles, using a Branson sonicator. Crude chromatin was centrifuged at 14000 rpm in a microcentrifuge at 4°C and supernatant was transferred to a 15-ml tube. An equal volume of room temperature 2X radioimmunoprecipitation assay (RIPA) buffer (2% Triton X-100, 280 mM NaCl, 20 mM Tris-HCl [pH 8.0], 2 mM EDTA) was added. The chromatin was precleared by adding 10 µl/ml of a 50% slurry containing an equal mixture of agarose beads coupled to protein A and protein G (Millipore; equal volume of protein A and G beads were mixed) previously washed three times with 1 X RIPA buffer and blocked 2 hr at room temperature with 0.1 mg/ml bovine serum albumin and 0.2 mg/ml salmon sperm DNA. For immunoprecipitations, 1 ml of precleared chromatin was incubated with 5 µl preimmune, 5 µl Rbf1 antibody [24], or 2 µl H3 antibody (Abcam, Cambridge, MA; 0.4 µg/ µl) overnight at 4°C. After overnight incubation, the samples were centrifuged at 14000 rpm in a microcentrifuge for 10 minutes at 4°C. Supernatant was taken in microcentrifuge tubes and 40 µl blocked beads (as described above) were added to each sample and incubated for 2 h (with rotating) at 4°C. The beads were centrifuged in a microcentrifuge at 1000 rpm for 1 min, washed twice with 1ml ice cold low-salt buffer (0.1% SDS, 1% Triton X-100, 2 mM EDTA, 20 mM Tris-Cl [pH 8.0], 150 mM NaCl), twice with ice cold high-salt buffer (0.1% SDS, 1% Triton X-100, 2 mM EDTA, 20 mM Tris-Cl [pH 8.0], 500 mM NaCl) and twice with Tris-EDTA (10 mM Tris [pH 8.0], 1 mM EDTA). Chromatin was eluted at room temperature with 250 µl elution buffer (1% SDS, 0.1 M $NaHCO_3$) for 15 min without shaking. Beads were centrifuged, the supernatant was transferred to a microcentrifuge tube, a second elution was performed, and supernatants were combined. 25 µl of 4M NaCl was added, and cross-links were reversed overnight at 65°C. The eluates were then incubated with 1 µl RNase A (10 mg/ml) at 37°C for 30 min. 10 µl 0.5 M EDTA, 20 µl 1 M Tris-Cl (pH 6.5), and 1 µl proteinase K (20 mg/ml) were then added and tubes incubated at 42°C for 1 h. DNA was extracted with phenol-chloroform and precipitated with equal volume of isopropanol, 3 M sodium acetate (final concentration 0.3M) and GlycoBlue pellet paint by centrifuging at 14000 rpm at 4°C. The pellets were carefully washed once with 70% ethanol, air dried and resuspended in 40 µl water. 2 µl of each ChIP sample was used for 31 cycles of PCR for 0-6 hr embryos and 28 cycles of PCR for the DNA from other time points. The oligonucleotides used for PCR are listed in Table S8.
